# Cutaneous Calcium/Calmodulin‐Dependent Protein Kinase II‐γ–Positive Sympathetic Nerves Secreting Norepinephrine Dictate Psoriasis

**DOI:** 10.1002/advs.202306772

**Published:** 2024-03-28

**Authors:** Yafen Yu, Weiwei Chen, Bao Li, Zhuo Li, Yirui Wang, Yiwen Mao, Wencheng Fan, Yuanming Bai, Hongbo Hu, Qi Zhen, Liangdan Sun

**Affiliations:** ^1^ Department of Dermatology the First Affiliated Hospital of Anhui Medical University Hefei 230032 China; ^2^ North China University of Science and Technology Affiliated Hospital Tangshan 063210 China; ^3^ Health Science Center North China University of Science and Technology Tangshan 063210 China; ^4^ School of Public Health North China University of Science and Technology Tangshan 063210 China; ^5^ Inflammation and Immune Diseases Laboratory of North China University of Science and Technology Tangshan 063210 China; ^6^ Key Laboratory of Dermatology Anhui Medical University Ministry of Education Hefei 230032 China; ^7^ The Center for Scientific Research the First Affiliated Hospital of Anhui Medical University Hefei 230032 China; ^8^ The Comprehensive Lab College of Basic Anhui Medical University Hefei 230032 China; ^9^ Center for Immunology and Hematology State Key Laboratory of Biotherapy West China Hospital Sichuan University Chengdu 610065 China

**Keywords:** adrenoceptor β1, alcaftadine, CAMK2γ, norepinephrine, psoriasis, sympathetic nerves, γδT cells

## Abstract

Cutaneous sympathetic nerve is a crucial part of neuropsychiatric factors contributing to skin immune response, but its role in the psoriasis pathogenesis remains unclear. It is found that cutaneous calcium/calmodulin‐dependent protein kinase II‐γ (CAMK2γ), expressed mainly in sympathetic nerves, is activated by stress and imiquimod in mouse skin. *Camk2g*‐deficient mice exhibits attenuated imiquimod‐induced psoriasis‐like manifestations and skin inflammation. CaMK2γ regulates dermal γδT‐cell interleukin‐17 production in imiquimod‐treated mice, dependent on norepinephrine production following cutaneous sympathetic nerve activation. Adrenoceptor β1, the primary skin norepinephrine receptor, colocalises with γδT cells. CaMK2γ aggravates psoriasiform inflammation via sympathetic nerve–norepinephrine–γδT cell–adrenoceptor β1–nuclear factor‐κB and –p38 axis activation. Application of alcaftadine, a small‐molecule CaMK2γ inhibitor, relieves imiquimod‐induced psoriasis‐like manifestations in mice. This study reveals the mechanisms of sympathetic‐nervous‐system regulation of γδT‐cell interleukin‐17 secretion, and provides insight into neuropsychiatric factors dictating psoriasis pathogenesis and new potential targets for clinical psoriasis treatment.

## Graphical abstract

Calcium/calmodulin‐dependent protein kinase II‐γ (CAMK2γ) is activated in imiquimod‐ or stress‐activated sympathetic nerves and increases the interleukin‐17 production of dermal γδT cells through the norepinephrine–γδT–adrenoceptor β1–nuclear factor‐κB and –p38 axes in the skin, resulting in excessive psoriatic inflammation. Alcaftadine, a small‐molecule compound that significantly inhibited CaMK2γ activity, effectively alleviated psoriasis symptoms.

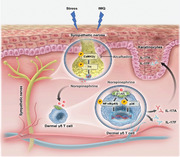

## 1 Introduction

Psoriasis is a chronic inflammatory skin disease characterized by immune‐cell infiltration, epidermal hyperproliferation, and abnormal keratinocyte differentiation.^[^ ^1^ ^]^ The development is driven by keratinocyte–immune‐cell interaction to produce a network of cytokines, particularly interleukin (IL)−17, and subsequent signaling cascades.^[^ ^2^, ^3^ ^]^ IL‐17, released by various cell types, including T helper (Th)17 cells, cytotoxic T17 cells, IL‐17–producing γδT (γδT17) cells, and group‐3 innate lymphoid cells,^[^ ^4^, ^5^, ^6^ ^]^ plays a crucial role in driving the recruitment of neutrophils and monocytes to psoriatic lesions.^[^ ^2^ ^]^ In skin inflammation, γδT cells are the major IL‐17 source,^[^ ^7^ ^]^ and have a significant contribution to imiquimod (IMQ)‐induced psoriasis development in mouse models.^[^ ^8^, ^9^ ^]^

For many patients with psoriasis, physical effects (e.g., itching, pain) are accompanied by neuropsychiatric symptoms such as anxiety and depression. Epidemiological studies have shown that neuropsychiatric factors play important roles in the onset and aggravation of psoriasis.^[^ ^10^ ^]^ Nicole Ward's research team substantiated the crucial involvement of nerves in psoriasis pathogenesis in both animal models and patients.^[^ ^11^, ^12^, ^13^ ^]^ Recent studies have uncovered that nociceptive sensory neurons, by dermal dendritic cells, regulate psoriasiform skin inflammation through the IL‐23/IL‐17 pathway.^[^ ^9^ ^]^ Epidural nerve block with lidocaine effectively alleviates psoriasis by inhibiting the IL‐23 production regulated by sensory nerve–dendritic cell interaction, which relies on the CGRP pathway.^[^ ^14^ ^]^ However, the regulatory effect of other neuropsychiatric factors on psoriasis, notably the involvement of neurotransmitters, remains inadequately comprehended.

The crucial neuroimmune communication pathway involves direct effects of sympathetic nervous system (SNS) activity on immune cells.^[^ ^15^ ^]^ SNS–immune‐system interaction has been found to regulate the occurrence of gastrointestinal, liver and other diseases.^[^ ^16^, ^17^, ^18^ ^]^ Nevertheless, the role of sympathetic nerves, which are widely distributed in skin, in skin diseases remains unclear. Norepinephrine (NE) serves as a prominent neurotransmitter in sympathetic neurons, and the sympathetic nerves, particularly adrenergic nerves in close proximity to T cells, are a primary source of NE in skin.^[^ ^19^ ^]^ Clinical studies have found that SNS activity in psoriatic lesions and serum NE levels are dysregulated in patients with psoriasis, indicating that the skin SNS plays an important role in psoriasis occurrence and development.^[^ ^20^ ^]^ But whether and how the cutaneous sympathetic nerves regulate inflammation in psoriasis remain unknown.

Psoriasis susceptibility genes involved in immune responses, cell proliferation and apoptosis, skin barrier protection, and other functions have been identified.^[^ ^1^, ^21^ ^]^ *CAMK2G* and *CAMK4* as candidate neurogenic psoriasis susceptibility genes, and demonstrated that *CAMK4* regulated psoriasis mainly through the monocyte–macrophage system.^[^ ^22^ ^]^ CAM*K2G* is a serine and threonine kinase of the calcium/calmodulin‐dependent protein kinase II (CaMKII) family that encodes CaMK2γ, which is involved in nerve‐cell development via the regulation of dendritic spine and synapse formation and neuronal plasticity.^[^ ^23^, ^24^, ^25^, ^26^ ^]^ Clinical case–control study has shown a statistically significantly reduced psoriasis risk for use verapamil, a calcium channel blocker.^[^ ^27^ ^]^ CaMKII family members have been implicated in catecholamine biosynthesis; they stimulate the phosphorylation of the rate‐limiting enzyme tyrosine hydroxylase (TH) in some neuronal populations.^[^ ^28^, ^29^ ^]^ CaMKII hyperactivity enhances presynaptic and postsynaptic N‐methyl‐D‐aspartate receptor activity to increase sympathetic vasomotor tone in hypertensive patients.^[^ ^30^ ^]^ CaMKII regulates the fight‐or‐flight SNS response induced by external stressors through the ARβ pathway.^[^ ^31^, ^32^ ^]^

Here, we show that CaMK2γ in skin is expressed mainly in sympathetic nerves and is strongly activated by restraint stress and IMQ. Restraint stress aggravates IMQ‐induced psoriatic inflammation, accompanied by increased CAMK2γ activity and abnormal sympathetic nerve activation. The activated CAMK2γ level is higher in human psoriatic lesion skin than in healthy skin. In an IMQ‐induced psoriasis model, *Camk2g^–/–^* mice exhibited significantly weakened psoriasis‐like manifestations and decreased γδT‐cell IL‐17A production relative to wild‐type (WT) mice. CaMK2γ increased the number of γδT17 cells in IMQ‐treated mice, dependent on NE production due to cutaneous sympathetic nerve activation. CaMK2γ^+^ sympathetic nerves regulated psoriasiform inflammation through the NE–γδT–ARβ1–nuclear factor (NF)‐κB and –p38 axes. The application of alcaftadine, a small‐molecule inhibitor targeting CaMK2γ, efficiently relieved IMQ‐induced psoriasis in mice. Thus, CaMK2γ and ARβ1 are potential targets for psoriasis treatment.

## 2 Result

### 2.1 Cutaneous CaMK2γ Expression

**Figure 1:**
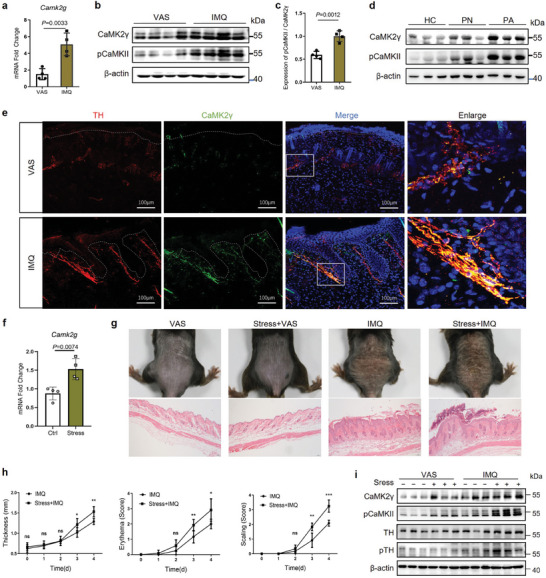
Cutaneous CaMK2γ is expressed mainly in sympathetic nerves and strongly activated in response to acute stress and IMQ application. a) *Camk2g* mRNA levels in mouse skin, determined by qPCR (*n* = 4). b) Western blots of CaMK2**γ** in mouse skin. c) The quantification of pCaMKII / total CaMK2**γ**. d) Western blots of CaMK2**γ** in healthy (HC, *n* = 3), non‐lesional (PN, *n* = 3) and psoriatic lesional skin (PA, *n* = 3). e) Representative immunofluorescent images of mouse skin sections stained for TH (sympathetic nerves, red), CaMK2γ (green), and DAPI (nuclei, blue). Scale bars = 100 µm. f) *Camk2g* mRNA levels in the skin of IMQ‐treated mice under tube restraint stress, determined by qPCR (*n* = 4). g) Representative photographs and hematoxylin and eosin (HE)–stained sections of mouse back skin on day 4 of VAS and IMQ treatment (*n* = 5). Scale bars = 50 µm. h) Scoring curves of back skin thickness, scaling, and erythema. i) Western blots of CaMK2**γ**, pCaMKII, TH, and pTH in mouse skin (*n* = 3). Data are representative of three independent experiments and shown as mean ± SD. a,c,f,h) Two‐tailed unpaired Student's *t* test. ns, not significant.

We examined cutaneous *CAMK2G* expression in psoriatic patients and mice. Real‐time quantitative polymerase chain reaction (qPCR) showed that the *Camk2g* transcript level in skin was significantly higher in mice with IMQ‐induced psoriasis than in Vaseline (VAS)‐treated controls (**Figure** **1**a). An RNA sequencing (RNA‐seq) dataset survey indicated that CaMK2γ was the predominant CaMKII isoform in psoriatic mouse skin (Figure S1a, Supporting Information). CaMK2γ activity was stimulated by direct Ca^2+^/CaM binding to regulatory domains. Phosphorylation at T287 is proved to be its activated form.^[^ ^33^ ^]^ Using phosphorylated (p)CaMKII (Thr287) antibody, we detected strong elevation of both total CaMK2γ and the activated CaMK2γ level in psoriatic mouse skin, and IMQ treatment markedly increased CaMK2γ phosphorylation (Figure 1b,c). We also found that the CaMK2γ protein level was highest in psoriatic lesional skin, followed by non‐lesional, and lowest in healthy skin (Figure 1d). CaMK2γ phosphorylation was highly elevated in psoriatic lesional skin compared to healthy skin (Figure S1b, Supporting Information).

Whole mount staining of both mouse skin tissues and human skin samples for CaMK2γ and the sympathetic nerves marker TH,^[^ ^34^ ^]^ showed that cutaneous CaMK2γ was expressed mainly in dermal sympathetic nerve fibers (Figure 1e; Figure S1c, Supporting Information). To determine whether increased CAMK2G expression in psoriasis is associated with SNS activation, we applied a tube restraint–induced acute stress model and found that acute stress indeed increased cutaneous *Camk2g* transcription (Figure 1f). Additionally, we found that acute stress aggravated the severity of IMQ‐induced psoriasis‐like manifestations, reflecting increased back skin thickness, aggravated erythema, and scaling (Figure 1g,h; Figure S1d,e, Supporting Information). The IMQ–tube restraint condition markedly increased CaMK2γ and TH phosphorylation and the cutaneous NE level, reflecting sympathetic nerve activation (Figure 1i; Figure S1f, Supporting Information). The pTH level was higher in human psoriatic lesional skin than in healthy skin (Figure S1g, Supporting Information). These findings suggests that sympathetic CaMK2γ was involved in the development of psoriasis.

### 2.2 *Camk2g* Deletion Attenuated Psoriasis

**Figure 2:**
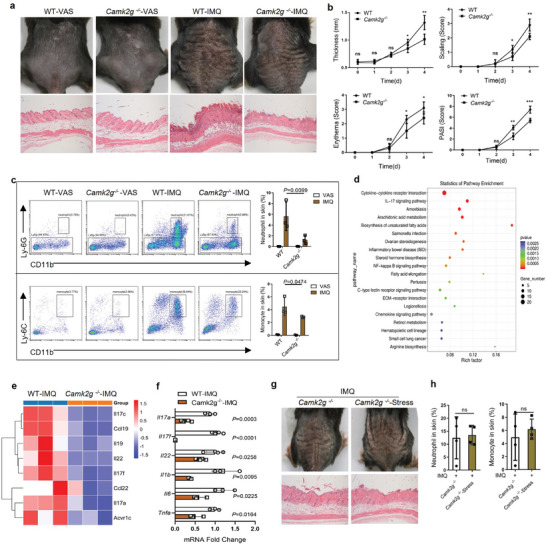
*Camk2g* deficiency attenuates psoriasis‐like manifestations and skin inflammation induced by IMQ. a) Representative photographs and HE–stained sections of mouse back skin on day 4 of VAS and IMQ treatment (*n* = 5). Scale bars = 50 µm. b) Scoring curves of back skin thickness, scaling, erythema, and PASI. c) Representative flow cytometric images and statistics for the percentage of total inflammatory monocytes and neutrophils in digested back skin (*n* = 3) on day 4. d) Kyoto Encyclopedia of Genes and Genomes pathway enrichment of differentially expressed genes of whole skin from IMQ‐treated WT and *Camk2g^–/–^* mice. e) Heat maps of differentially expressed genes in whole skin, obtained by RNA‐seq (*n* = 3). f) Whole‐skin mRNA levels of proinflammatory genes in IMQ‐treated mice, determined by qPCR (*n* = 3–4). g) Representative photographs and hematoxylin and eosin (HE)–stained sections of mouse back skin on day 4 of IMQ treatment (*n* = 5). Scale bars = 50 µm. h) The percentage of total inflammatory monocytes and neutrophils in digested back skin (*n* = 4) on day 4. Data are representative of three independent experiments and shown as mean ± SD. b,f,h) two‐tailed unpaired Student's *t* test. c) Two‐way analysis of variance. *p < 0.05, **p < 0.01, ***p < 0.001; ns, not significant.

To test the mechanism of CaMK2γ in psoriasis, we generated IMQ‐induced psoriasis mouse model of *Camk2g* deficiency (*Camk2g* ^−/−^) mice and WT mice. Compared to IMQ‐treated WT mice, IMQ‐treated *Camk2g* ^–/–^ mice displayed significantly weakened psoriasis‐like manifestations, as indicated as reduced back skin thickness, mitigated erythema, and scaling (**Figure** **2**a,b). We used specific labeled antibodies to cluster monocytes and neutrophils, which are typical inflammatory infiltrating cells in psoriatic lesions from mouse back skin.^[^ ^2^ ^]^ revealed significantly less IMQ‐induced inflammatory infiltration in *Camk2g* ^–/–^ than in WT mice (Figure 2c). The gating strategies were shown in Figure S2a (Supporting Information).

To fully understand the transcriptional characteristics of IMQ‐treated WT and *Camk2g* ^−/−^ mice, we performed whole‐skin RNA sequencing and identified genes expressed differentially between IMQ‐treated WT and *Camk2g* ^–/–^ mice, including those related to cytokine–cytokine receptor interaction and the IL‐17 signal pathway (Figure 2d,e). qPCR revealed significantly reduced expression of proinflammatory genes known to be expressed more strongly in psoriatic lesions than in healthy skin (*Il17a*, *Il17f*, *Il6*, *Il22*, *Il1b*, and *Tnfa*) in skin from IMQ‐treated *Camk2g* ^–/–^ mice^[^ ^3^ ^]^ (Figure 2f). It is further suggested that *Camk2g* deletion attenuated IMQ‐induced psoriatic skin inflammation in mice. Notably, we applied IMQ‐tube restraint treatment to *Camk2g* ^−/−^ mice, and found that acute stress did not aggravate the IMQ‐induced psoriatic phenotype and inflammatory infiltration in *Camk2g* deficient mice (Figure 2g,h). This further suggested that the regulation of SNS in psoriasis depends on CaMK2γ.

### 2.3 CaMK2γ Regulated γδT‐Cell IL‐17A Release

**Figure 3:**
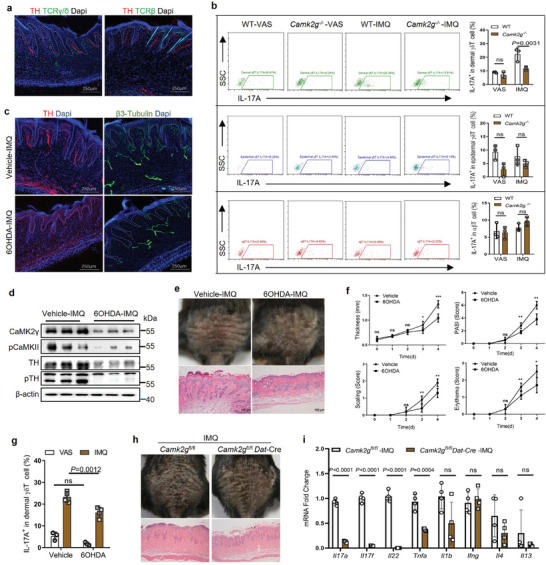
CaMK2γ promotes IL‐17 production by dermal γδT cells via cutaneous sympathetic nerve activation. a) Representative immunofluorescent images of mouse skin sections stained for TH (red), TCRγ/δ (green), or TCRβ (green) and DAPI (nuclei, blue). Scale bars = 250 µm. b) Representative flow cytometric images and statistics for the percentage of IL‐17A^+^ cells among dermal and epidermal γδT cells and αβT cells from digested back skin (*n* = 3). c) Representative immunofluorescent images of vehicle‐ and 6‐OHDA–treated mouse skin sections stained for TH (red), β3‐Tubulin (peripheral nerves, green), and DAPI (blue) (*n* = 5). Scale bars = 250 µm. d) Western blots of CaMK2γ, pCaMKII, TH, and pTH in vehicle‐ and 6‐OHDA–treated mouse skin on day 4 of IMQ treatment (*n* = 3). e) Representative photographs of back skin and HE–stained mouse skin sections on day 4 of IMQ treatment (*n* = 5). Scale bars = 100 µm. f) Scoring curves of back skin thickness, scaling, erythema, and PASI (*n* = 5). g) Representative flow cytometric images and statistics for the percentage of IL‐17A+ cells among dermal γδT cells from digested back skin (*n* = 3–4). h) Representative photographs of back skin and HE–stained skin sections from *Camk2g^fl/fl^* and *Camk2g^fl/fl^ Dat‐*Cre mice on day 4 of IMQ treatment (*n* = 5). Scale bars = 50 µm. i) Whole‐skin mRNA levels of proinflammatory genes in IMQ‐treated mice, determined by qPCR (*n* = 4). Data are representative of three independent experiments and shown as mean ± SD. b,g) Two‐way analysis of variance. f,i) two‐tailed unpaired Student's *t* test. *p < 0.05, **p < 0.01, ***p < 0.001; ns, not significant.

In the back skin of IMQ‐treated WT mice, IL‐17A was produced mostly by dermal γδT cells and rarely expressed in epidermal γδT and αβT cells (Figure S2b,c, Supporting Information). The whole mounts staining showed that in the skin tissues of IMQ‐treated mice, γδT cells were in closer proximity to sympathetic fibers than αβT cells (**Figure** **3**a). Flow cytometry revealed markedly fewer IL‐17A^+^ dermal γδT cells in IMQ‐treated *Camk2g^–^* ^/–^ mice than in WT counterparts, but no difference in the number of IL‐17A^+^ epidermal γδT or αβT cells (Figure 3b; Figure S2d, Supporting Information). Thus, cutaneous sympathetic CaMK2γ may regulate local IL‐17A production by mainly dermal γδT cells.

6‐hydroxydopamine (6‐OHDA) is a selective catecholaminergic neurotoxin which is used to ablate the sympathetic nerve. The intraperitoneally injection of 6‐OHDA has been found to reduce IMQ‐induced ear swelling.^[^ ^9^ ^]^ To survey the effect of skin local sympathetic nerves ablation on psoriatic mice, we adopted an intradermal injection of 6‐OHDA before IMQ application and found that it could specifically ablate the cutaneous sympathetic nerves (Figure 3c). Local CaMK2**γ^+^** sympathetic nerve inactivation was confirmed by significant decreases in activated pCaMKII and pTH in the skin (Figure 3d). Cutaneous sympathectomy alleviated psoriasis‐like manifestations in IMQ‐treated mice (Figure 3e,f). Sympathectomized skin contained fewer IL‐17A^+^ γδT cells, indicating that cutaneous sympathetic nerves play a role in skin inflammation by regulating γδT‐cell IL‐17 production (Figure 3g).

To directly evaluate the function of sympathetic nerve‐specific *Camk2g* in psoriasis, we generated a *Camk2g* gene conditional knockout mouse (*Camk2g* ^fl/fl^ *Dat*‐Cre). The results showed that psoriatic symptoms were alleviated, γδT‐cell IL‐17A production was reduced and mRNA levels of *Il17a*, *Il17f*, *Il22*, and *Tnfa* (but not *Il1b*, *Ifng*, *Il4*, or *Il13*) were markedly reduced in IMQ‐treated *Camk2g* ^fl/fl^ *Dat*‐Cre mice relative to IMQ‐treated controls (Figure 3h,i; Figure S3a–c, Supporting Information). Thus, CaMK2**γ^+^** sympathetic nerves may control IMQ‐induced cytokine production by γδT17 cells, rather than IFN‐γ–producing (e.g., γδT1) or IL‐4–producing (e.g., Th2) cells.

### 2.4 IL‐17 Control by CaMK2γ Depended on NE

**Figure 4:**
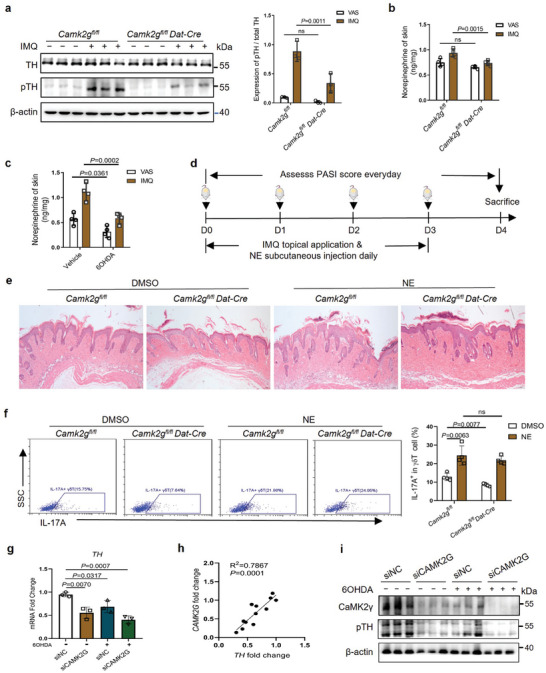
CaMK2γ’s regulation of the IMQ‐induced IL‐17 production of γδT cells depends on the secretion of norepinephrine. a) Western blots and quantification of CaMK2γ, pCaMKII, TH, and pTH in mouse skin on day 4 of IMQ treatment (*n* = 3). b,c) NE concentrations in the skin of *Camk2g^fl/fl^* and *Camk2g^fl/fl^ Dat‐Cre* mice (b, *n* = 5) and vehicle‐ and 6‐OHDA–treated mice (c, *n* = 4) on day 4 of VAS and IMQ treatment, determined by enzyme‐linked immunosorbent assay (ELISA). d) Flow chart of NE injection in the IMQ‐induced mouse model. e) Representative HE–stained skin sections from *Camk2g^fl/fl^* and *Camk2g^fl/fl^ Dat‐Cre* mice injected subcutaneously with DMSO and NE on day 4 of IMQ treatment (*n* = 5). Scale bars = 50 µm. f) Frequency of IL‐17A^+^ cells among γδT cells from DMSO‐ and NE‐treated mouse skin (*n* = 4). g) *TH* mRNA levels in 6‐OHDA‐treated SH‐SY5Y cells, determined by qPCR (*n* = 3). h) The relationships between the expression of *TH* and *CAMK2G* in SH‐SY5Y cells (*n = 12*). i) Western blots of CaMK2γ and pTH in 6‐OHDA–treated SH‐SY5Y cells (*n* = 3). Data are representative of three independent experiments and shown as mean ± SD. a,b,c,f) Two‐way analysis of variance. g) two‐tailed unpaired Student's *t* test. h) linear regression analysis. ns, not significant.

IMQ treatment significantly upregulated TH phosphorylation in mouse skin, and sympathetic nerve‐specific *Camk2g* deletion attenuated this effect (**Figure** **4**a). Catecholamine neurotransmitter detection revealed lower skin NE levels in IMQ‐treated *Camk2g* ^fl/fl^ *Dat*‐Cre mice than in WT counterparts (Figure 4b). The genetic deletion of *Camk2g* decreased both skin and serum NE level in IMQ‐treated mice (Figure S4a,b, Supporting Information). The serum NE level was higher in patients with psoriasis than in healthy controls (Figure S4c, Supporting Information). NE reduction in sympathectomized skin implied that the sympathetic nerves’ regulation of γδT‐cell IL‐17 production depended on NE secretion (Figure 4c). In IMQ‐treated mice, subcutaneous NE injection aggravated psoriasis‐like manifestations, eliminated the difference in skin thickness between WT and *Camk2g* ^fl/fl^ *Dat*‐Cre mice, increased the number of IL‐17A^+^ γδT cells in skin and rescued the *Camk2g* deletion–induced reduction of this number (Figure 4d–f; Figure S4d,e, Supporting Information). Thus, the effect of CaMK2γ on IMQ‐induced skin inflammation may be related to NE secretion from cutaneous sympathetic nerves.

qPCR showed that *CAMK2G* knockdown by RNA interference and 6‐OHDA–induced injury inhibited TH transcription in PMA‐differentiated human neuroblastoma (SH‐SY5Y) cells, which constitutively express TH and are morphologically, physiologically and biochemically similar to adrenergic neuron^[^ ^35^ ^]^ (Figure 4g). The transcription of *TH* was positively correlated with *CAMK2G* transcription (Figure 4h). Western blotting showed that 6‐OHDA–induced injury reduced the CaMK2γ and pTH levels in these cells (Figure 4i). *CAMK2G* deletion significantly decreased the activated pTH level (Figure 4i). These findings suggest that CaMK2γ promoted NE secretion in skin by activating TH in cutaneous sympathetic nerves, with subsequent upregulation of γδT‐cell IL‐17A production.

### 2.5 Role of ARβ1–NF‐κB and –p38 Signaling

**Figure 5:**
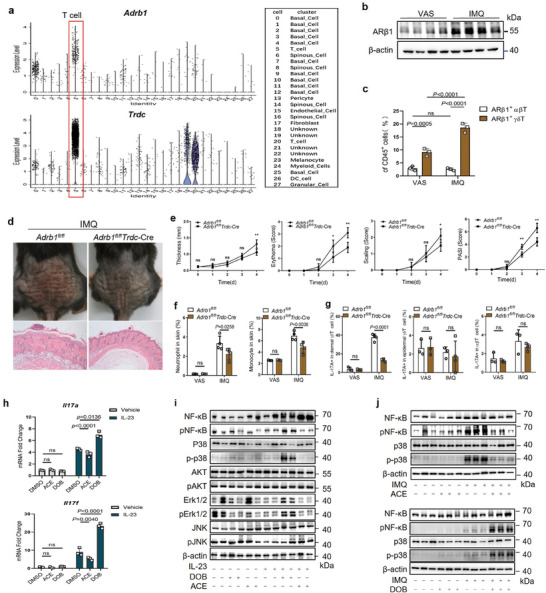
Cutaneous sympathetic nerves mediate γδT‐cell release of IL‐17A through ARβ1–NF‐κB and ARβ1‐p38 signalling. a) Expression of *Adrb1* and *Trdc* in VAS‐ and IMQ‐treated psoriatic murine skin, determined by 10X single‐cell sequencing (*n* = 3). b) Western blots of ARβ1 in mouse skin (*n* = 3). c) Frequency of ARβ1^+^ T‐cell subsets among CD45^+^ cells from digested murine back skin, determined by flow cytometry (*n* = 4). d) Representative photographs and HE–stained back skin sections from *Adrb1^fl/fl^* and *Adrb1^fl/fl^ Trdc‐Cre* mice on day 4 of IMQ treatment. Scale bars = 100 µm. e) Scoring curves of back skin thickness, scaling, erythema, and PASI (*n* = 5). f) Frequencies of monocytes and neutrophils among CD45^+^ cells from digested back skin, determined by flow cytometry (*n* = 4). g) Frequencies of IL‐17A^+^ cells among dermal and epidermal γδT cells and αβT cells from digested back skin (*n* = 4). h) mRNA levels of *Il17a / Il17f* in IL‐23–activated γδT cells treated with ACE or DOB, determined by qPCR (*n* = 3). i,j) Western blots of relative protein expression in IL‐23–stimulated γδT cells (i) and IMQ‐treated mice (j) treated with DOB or ACE (*n* = 3). Data are representative of three independent experiments and shown as mean  ±  SD. c,f,g,h) Two‐way analysis of variance. *p < 0.05, **p < 0.01, ***p < 0.001; ns, not significant.

NE is considered to be mainly a selective adrenergic receptor (AR) β1 agonist.^[^ ^36^ ^]^ Single‐cell sequencing showed that *Adrb1* was expressed mainly in T cells in psoriatic mouse skin (**Figure** **5**a). While the genes encoding other adrenoceptors were poorly or unspecifically expressed in T cells (Figure S5a, Supporting Information). The expression of ARβ1 was increased in IMQ‐treated relative to VAS‐treated control mouse skin (Figure 5b). Flow cytometry showed that IMQ application increased the number of ARβ1^+^ γδT cells but not ARβ1^+^ αβT cells in skin‐infiltrating immune cells and the number of ARβ1^+^ γδT cells was higher than that of ARβ1^+^ αβT cells in both IMQ‐treated and VAS‐treated mouse skin (Figure 5c). Immunohistochemistry and western blotting showed that ARβ1 was expressed on dermis‐infiltrating immune cells and that expression was increased in human psoriatic lesional skin relative to healthy skin (Figure S5b,c, Supporting Information). ARβ1 was expressed on γδT cells and the number of ARβ1^+^ T cells was increased in human psoriatic lesional skin relative to healthy skin (Figure S5d,e, Supporting Information). These data indicate that NE–ARβ1 signaling may have an effect on γδT17 cells.

Topical application of the selective ARβ1 agonist dobutamine hydrochloride (DOB) to IMQ‐treated mice aggravated psoriasis‐like manifestations and promoted γδT‐cell IL‐17A production in skin (Figure S6a–d, Supporting Information); application of the selective ARβ1 antagonist acebutolol hydrochloride (ACE) had the opposite effects (Figure S6e–g, Supporting Information).

To investigate whether ARβ1 effects on γδT17 cells directly, we deleted *Adrb1* from the γδT cells by using *Trdc*‐Cre. The qPCR analysis demonstrated the effective knockout of *Adrb1* in γδT cells from *Adrb1* ^fl/fl^ *Trdc*‐Cre mice relative to *Adrb1* ^fl/fl^ controls. Nevertheless, there were no significant changes observed in the CD4^+^ T and CD8^+^ T cells. (Figure S6h, Supporting Information). Relative to IMQ‐treated *Adrb1* ^fl/fl^ control mice, IMQ‐treated *Adrb1* ^fl/fl^ *Trdc*‐Cre mice had significantly reduced back‐skin thickness, erythema, scaling and total Psoriasis Area and Severity Index (PASI) scores, and inflammatory infiltration and IL‐17^+^ dermal γδT cells (Figure 5d–g). Mouse γδT‐cell deficiency (*Tcrd^–/–^*) counteracted subcutaneously injected DOB's promotion of the psoriatic phenotype, inflammatory infiltration and αβT‐cell IL‐17A production (Figure S6i,j, Supporting Information). In addition, we injected IMQ‐treated mice intraperitoneally with the ARβ1 antagonist (ACE) and the highly selective ARβ2 antagonist zenidolol hydrochloride (ZEN), respectively. The findings showed that systemic ARβ1 inhibition also alleviated the psoriatic phenotype in mice and decreased the expression of IL‐17A in γδT‐cells in the skin (Figure S7a–d, Supporting Information). However, inhibiting ARβ2 activity did not change the phenotype of psoriatic mice but led to increased neutrophil infiltration in the mice's skin (Figure S7e–h, Supporting Information). Thus, ARβ1 activation and inhibition respectively promoted and reduced γδT‐cell IL‐17A production. Cutaneous sympathetic nerves mediated IL‐17 release by γδT, but not αβT, cells through NE–ARβ1 signaling.

In γδT cells subjected to magnet‐activated sorting, ACE and DOB respectively reduced and increased IL‐17A and IL‐17F production under IL‐23 stimulation (Figure 5h). Quantitative proteomics revealed Th17 cell differentiation, related gene (e.g., NF‐κB inhibitor ε) upregulation and MAPK signaling in DOB‐activated γδT cells (Figure S8a,b, Supporting Information). In IL‐23–activated γδT cells, DOB markedly increased p38 and NF‐κB phosphorylation, and ACE had the opposite effect, but neither influenced the activation of the MAPKs extracellular signal‐regulated kinase (ERK) and c‐Jun N‐terminal kinase (JNK; Figure 5i). Thus, ARβ1's regulation of T‐cell activation depended on p38 and NF‐κB, not ERK or JNK, phosphorylation. IMQ exposure activated p38 and NF‐κB p65 phosphorylation in mouse skin; DOB enhanced this effect and ACE inhibited it (Figure 5j; Figure S8c,d, Supporting Information). Furthermore, NE treatment markedly increased IL‐17A protein levels in IL‐23‐activated γδT cells. While the inhibition of p38 using SB202190 or NF‐κB using SC75741 both blocked this increase (Figure S8e, Supporting Information). These results suggest that CaMK2γ promotes T‐cell IL‐17 production via NE–ARβ1–p38 and –NF‐κB pathway activation. Taken together, the data indicate that CaMK2γ promotes cutaneous sympathetic nerve NE synthesis by activating TH. NE secretion from CaMK2γ^+^ sympathetic nerves enhance γδT‐cell IL‐17 production via the NE–ARβ1–p38 and –NF‐κB pathways to aggravate psoriasiform skin inflammation.

### 2.6 Alcaftadine Reduced Inflammation in Mice

**Figure 6:**
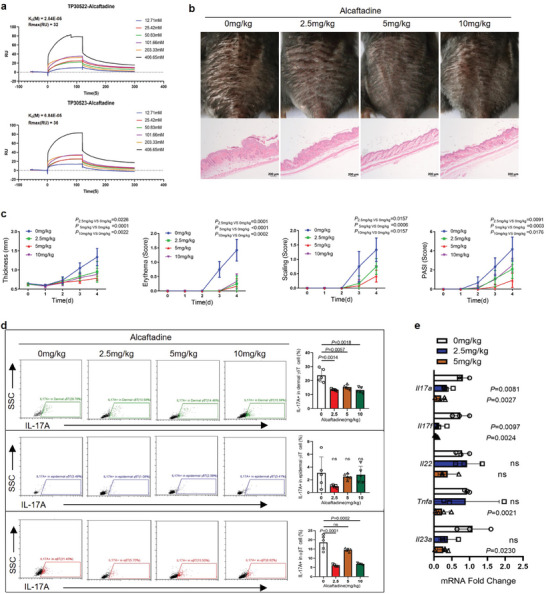
Alcaftadine, a small‐molecule inhibitor targeting CaMK2γ, effectively attenuates the inflammatory phenotype in IMQ‐treated mice. a) Affinity of alcaftadine for human (upper) and mouse (lower) CaMK2γ proteins. b) Representative photographs and HE–stained sections of back skin from alcaftadine‐treated mice on day 4 of IMQ treatment (*n* = 6). Scale bars = 100 µm. c) PASI scores (*n* = 6). d) Representative flow cytometric images and statistics of IL‐17A^+^ dermal and epidermal γδT cells and αβT cells from digested back skin (*n* = 3). e) Whole‐skin mRNA levels of proinflammatory genes in IMQ‐ and alcaftadine‐treated mice, determined by qPCR (*n* = 3). Data are representative of three independent experiments and shown as mean ± SD. c,e) Two‐tailed unpaired Student's *t* test. d) Two‐way analysis of variance. ns, not significant.

Given the important role of CaMK2γ in the pathogenesis of psoriasis and that effective inhibitors targeting CaMK2γ have not been reported, we are committed to finding an effective small‐molecule inhibitor of CaMK2γ for psoriasis treatment and to determine its therapeutic effect on psoriasis. Among 130 screened small‐molecule compounds, alcaftadine most significantly inhibited CaMK2γ activity. The molecular formula of the compound is C_19_H_21_N_3_O. The structure and synthetic routes are shown in Figure S9a,b (Supporting Information). The constants for alcaftadine's specific binding to human and murine CaMK2γ were 2.54 and 6.84 × 10^−5^ m, respectively, suggesting strong binding ability and inhibitory effects in both cases (**Figure** **6**a). Alcaftadine has a strong hydrophobic interaction with multiple hydrophobic amino acids and the interactions jointly maintain the binding of alcaftadine to the CAMK2γ protein (Figure S9c,d, Supporting Information). Topical alcaftadine application inhibited CaMK2γ phosphorylation and reduced the PASI score (*p *< 0.001) and proportion of γδT17 cells in skin lesions in IMQ‐treated mice relative to controls (Figure 6b–d; Figure S9e, Supporting Information). qPCR revealed significantly reduced *Il17a*, *Il17f*, *Tnfa*, and *Il23a* expression in skin from mice administered 5 mg kg^−1^ alcaftadine, but no change in *Tnfa* or *Il23a* expression in skin from mice administered 2.5 mg kg^−1^ alcaftadine (Figure 6e). Thus, 5 mg kg^−1^ alcaftadine significantly inhibited psoriasis. Mice treated with 5 and 10 mg kg^−1^ alcaftadine exhibited reduced body weight loss compared to the control group, suggestive of the compound's low toxicity (Figure S9f, Supporting Information). These findings support the potential safety profile of alcaftadine as a clinical agent for psoriasis.

## 3 Conclusion

In the present study, we found that the susceptibility gene *CAMK2G*, which is mainly expressed in cutaneous sympathetic nerves, affected the progression of psoriasis by promoting γδT‐cells IL‐17 production through norepinephrine secretion. The mechanism of sympathetic neuroendocrine regulation in the pathogenesis of psoriasis was analyzed. Psoriasis is a disease with a severe genetic predisposition. A significant progress in psoriasis research has been the discovery of various genetic psoriasis loci, which provides the basis for the above‐mentioned genetic susceptibility. The precise functions and roles of these susceptibility genes in corresponding target cell types are just beginning to emerge. To identify psycho‐neurological factors that regulate the pathogenesis of psoriasis, we explored associations of neurogenic susceptibility gene *CAMK2G* expression with psoriasis in clinical samples and animal models. We observed CAMK2γ activity, mainly in sympathetic nerves of skin, an increased in human and mouse psoriatic skin. SNS exerts a “fight or flight” response in the periphery through the release of NE, which plays an important role in regulating stress responses. TH, the rate‐limiting enzyme in the biosynthesis of catecholamines, including dopamine, norepinephrine, and epinephrine, was used to identify the sympathetic nerves in skin.^[^ ^34^ ^]^ The over‐activation of TH by Ser40‐phosphorylation is a key marker of SNS hyperactivation.^[^ ^37^ ^]^ The positive linear correlation between *TH* and *CAMK2G* expression in human neuroblastoma cell line suggested that CAMK2γ is closely related to SNS activity. Tube restraint–induced stress increased the pTH and NE levels and CAMK2γ activity in mouse skin, reflecting abnormal cutaneous sympathetic nerve activation and aggravating psoriatic inflammation. These findings suggest that the relationship between CAMK2γ and cutaneous SNS activity plays a role in the pathogenesis of psoriasis. Previous studies suggested that stress activates the hypothalamic–pituitary–adrenal and sympathetic adrenal medullary axes, causing neuroendocrine hormone cascade and proinflammatory cytokine release.^[^ ^38^ ^]^ SNS dysregulation accompanied by unresolved immune activation leads to cyclic, cascading hyper‐sympathetic nerve activity with systemic and local immune activation in some autoimmune diseases.^[^ ^39^ ^]^ SNS dysfunction precedes rheumatoid arthritis development^[^ ^40^ ^]^ and SNS activity worsens immune‐mediated arthritis severity via Th1 and Th17 immune responses.^[^ ^41^ ^]^ Patients with type 1 diabetes exhibit selective pancreatic‐islet sympathetic nerve loss.^[^ ^42^ ^]^ Psoriasis also has the classic features of SNS dysregulation, but the mechanism by which SNS activity influences it remains to be studied.

In the present study, we found that *Camk2g* deletion reduced the IMQ‐induced psoriatic phenotype, skin inflammation and cutaneous γδT17 cell number and NE level in mice. Exogenous NE administration and cutaneous sympathetic nerve ablation had opposite effects on γδT17 cells. Using sympathetic nerve–specific *CAMK2G* knockout mouse models, we demonstrated that the sympathetic nerves regulate NE secretion through CaMK2γ‐dependent TH phosphorylation and promotion of γδT‐cell IL‐17A production. In addition, inhibition of calcium channels to control hypertension reduces the severity and incidence of psoriasis also suggests a positive correlation between calcium signaling and the disease.^[^ ^27^ ^]^

Catecholamine affects the balance among Th cell subsets. Activated Th cells differentiate into effector and memory cell subsets, such as Th1, Th2, and Th17 cells, which express the marker cytokines interferon (IFN)‐γ, IL‐4, and IL‐17A, respectively. NE receptors on T‐cell surfaces respond to catecholamines.^[^ ^43^, ^44^ ^]^ In vitro, NE supplementation drives Th17 T‐lymphocyte polarization, as indicated by increased IL‐17A and IL‐22 expression.^[^ ^45^ ^]^ In the pathogenesis of psoriasis, IL‐17 and IL‐22 secreted by cutaneous γδT cells induce inflammatory cell recruitment in the skin and abnormal keratinocyte proliferation.^[^ ^46^, ^47^, ^48^, ^49^ ^]^ In this study, we elucidated the mechanism that catecholamines directly affect another type 17 immune response of γδT cells, but not Th17 cells. Restraint stress and cutaneous sympathectomy increased and decreased the NE levels in the IMQ‐treated skin, respectively. There are several kinds of NE receptors, dividing into alpha‐ and beta‐receptor.^[^ ^50^ ^]^ We found that ARβ1 was ARβ1 is the only one of all NE receptors specifically expressed on cutaneous γδT17 cells and more strongly in psoriatic lesions. In vivo and in vitro, an ARβ1 agonist and antagonist enhanced and inhibited γδT‐cell IL‐17 production, respectively. The agonist's promotion of the psoriatic phenotype and inflammatory infiltration was counteracted in *Tcrd* ^–/–^ mice. These results confirm that NE participates in the regulation of IL‐17 expression by binding to ARβ1 on γδT‐cell surfaces. p38 phosphorylation has been reported to regulate T‐cell activation and differentiation by promoting the IL‐17 expression in the peripheral immune system.^[^ ^51^, ^52^ ^]^ NF‐κB is required for the development of pro‐inflammatory Th17 cells. Rel/NF‐κB drives Th17 differentiation by binding to and activating two Rorg promoters that control RAR‐related orphan nuclear receptor (ROR)γT and RORγ expression.^[^ ^53^, ^54^ ^]^ We found that the regulation of ARβ1 on γδT17 cells also depends on p38 and NF‐κB activation.

We observed an interesting phenomenon, in which both topical application and intraperitoneal injection of ARβ1 antagonist alleviated the psoriatic phenotype and inflammatory infiltration in mice, whereas ARβ2 antagonist showed no effect on the psoriatic phenotype and the levels of IL‐17 expressed by γδT cells, and even increased neutrophil infiltration in the skin. We speculated that this might be because ARβ2 is not specifically expressed on γδT cells, and its systemic inhibition exerted different effects from ARβ1 activity inhibition through alternative pathways. Several case reports have suggested an association between the use of antihypertensive drugs targeting β‐adrenergic receptors and an increased risk of psoriasis, which may be associated with the selectivity of β‐blockers. Antagonists targeting different ADRB subtypes potentially exert varying effects on psoriasis initiation and exacerbation. Many epidemiological studies have shown that vascular diseases, including hypertension, are risk factors for psoriasis, and calcium channel blocker targeting hypertension can reduce the severity and incidence of psoriasis.^[^ ^1^, ^27^ ^]^ These different aspects of clinical studies provide us with a comprehensive reference for the pathogenesis of psoriasis. Our systematic research demonstrated that inhibition of the CaMK2γ‐ARβ1 axis can effectively improve the symptoms of psoriasis.

Finally, the CaMK2γ inhibitor alcaftadine efficiently relieved IMQ‐induced psoriasis in mice. The target point of alcaftadine for binding inhibition in psoriasis is located at upstream of an CaMK2γ pathway. Compared with the traditional biological inhibitors which usually act on a middle and a downstream psoriatic immune‐inflammatory pathway, alcaftadine may have a more considerable long‐term therapeutic effect. Moreover, most of traditional biological agents are system injection drugs, which are easy to affect other systems. However, alcaftadine is easy to absorb for external use, which can avoid many side effects caused by system administration, and has advantages of high safety, wide applicable population, convenient administration, and good compliance.

**Figure 7:**
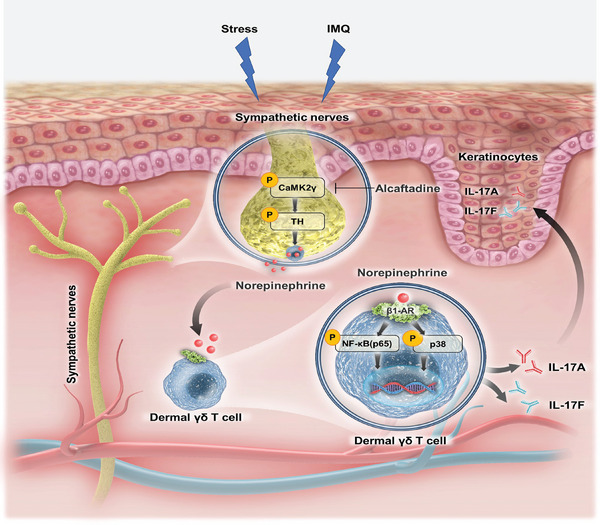
Diagram of the mechanism underlying the participation of cutaneous CAMK2γ^+^ sympathetic nerves in the pathogenesis of psoriasis. CaMK2γ is activated in IMQ‐ or stress‐activated sympathetic nerves and increases the IL‐17 production of dermal γδT cells through the NE–γδT–ARβ1–NF‐κB and –p38 axes in the skin, resulting in excessive psoriatic inflammation.

In summary, our study revealed a mechanism by which the SNS regulates γδT‐cell IL‐17 secretion and skin immune responses, providing a theoretical basis for neuropsychiatric factors’ psoriasis induction and aggravation. The over‐activated CaMK2γ promote the skin sympathetic nerves to secrete NE by activating of TH. NE then acts on γδT cells to promote IL‐17 production through the ARβ1–NF‐κB and –p38 axes (**Figure** **7**). The results suggest that *CAMK2G* and *ADRB1* are targets for the clinical treatment of psoriasis, especially that affected by neuropsychiatric factors. They open a new avenue for the treatment of inflammatory diseases in the skin, and perhaps elsewhere.

## 4 Experimental Section

### Human Participants

Patients with psoriasis vulgaris diagnosed by two senior dermatologists and healthy controls were recruited randomly and provided written informed consent. The exclusion criteria are listed in Table S1 (Supporting Information). Peripheral blood and skin tissue samples were collected at the Department of Dermatology and Cooperative Department of the First Affiliated Hospital of Anhui Medical University, Anhui, China, and processed according to the experimental requirements Tables S2 and S3, Supporting Information).

### Mice

Wild‐type, *Camk2g* ^–/–^, *Adrb1* ^fl/fl^, and *Trdc*‐Cre mice on a C57BL/6 background were purchased from GemPharmatech Corporation (Nanjing, China). *Camk2g* ^fl/fl^ and *Dat*‐Cre mice on a C57BL/6 background were purchased from Cyagen Corporation (Suzhou, China). *Tcrd^–/–^* mice was a gift from Professor Hu Hongbo of Sichuan University. All mice were housed under specific pathogen–free conditions on a 12/12‐h light/dark cycle at 21–25 °C and 30–75% humidity at the experimental animal centre of Anhui Medical University. Eight‐ to 10‐week‐old mice were used in the experiments.

### Animal Experiments

*Psoriasis Mouse Model Establishment*: The back hair of 7‐week‐old C57/BL6 mice was shaved to expose a skin area of 2 × 2 cm. Imiquimod (IMQ) cream (5%, 62.5 mg, Med‐shine Pharma #H20030129) or control Vaseline (VAS) cream (Lircon, Shandong, China) was applied topically to the exposed skin for 4 consecutive days. The severity of the psoriasis‐like phenotype on the back skin was assessed daily using the clinical Psoriasis Area and Severity Index (PASI). The PASI score was the sum of thickness, scaling, and erythema scores. The skin thickness was measured using a vernier caliper and scored on a scale ranging from 0 to 4 according to the standard shown in Table S4 (Supporting Information). Scaling and erythema were scored on a scale ranging from 0 to 4 (0, none; 1, slight; 2, moderate; 3, marked; 4, very marked). On day 5, the mice were sacrificed. Blood and skin samples were harvested for analysis.

*Stress Mouse Model Establishment*: Acute restraint stress was induced by keeping the mice in a chamber ventilated with a 50‐mL Falcon tube for 4 h. IMQ was applied to the backs of the mice thereafter.

*Subcutaneous Norepinephrine Injection*: For subcutaneous norepinephrine (NE, MedChemExpress #HY‐13715A) injection, a solution of 10 mg ml^−1^ NE was prepared freshly by dissolving NE in 3% dimethyl sulfoxide (DMSO, Sigma #D2650) in 1× phosphate‐buffered saline (PBS). The solution (50 µl) was injected subcutaneously into each mouse back 1 h before IMQ application once a day for 4 days. Control mice were injected with 50 µl vehicle (3% DMSO in PBS).

*Cutaneous Sympathetic Nerve Ablation*: A solution of 6 mg ml^−1^ 6‐hydroxydopamine hydrobromide (6‐OHDA, MedChemExpress #HY‐B1081A) was prepared freshly by dissolving 6‐OHDA in 0.1% ascorbic acid (MedChemExpress #HY‐B0166) in 1× PBS for intradermal injection. The solution (100 µl) was injected into mouse back skin 2 days before IMQ treatment. Control animals were injected with vehicle (100 µl PBS with 0.1% ascorbic acid).

*Topical Alcaftadine Application*: Three dose of alcaftadine (2.5, 5, 10 mg kg^−1^ body weight, Targetmol #T2533) dissolved in 150 µl vehicle (10% DMSO, 20% sulfobutylether‐β‐cyclodextrin) and 70% 1× PBS was applied topically to shaved mouse back skin 1 h before IMQ treatment once a day for 4 days. Vehicle without DOB was applied to control mice.

*Topical Acebutolol Application*: Acebutolol (ACE, 20 mg kg^−1^ body weight, MedChemExpress #HY‐17497) dissolved in VAS cream (Lircon, 10 mg drug/1 g cream) was applied topically to shaved mouse back skin 1 h before IMQ treatment once a day for 4 days. VAS cream without ACE was applied to control mice.

*Topical Dobutamine Application*: Dobutamine (DOB, 0.2 mg, 10 mg kg^−1^ body weight, MedChemExpress #HY‐15746) dissolved in 150 µl vehicle (10% DMSO, 20% sulfobutylether‐β‐cyclodextrin, Sangon Biotech #A600388) and 70% 1× PBS was applied topically to shaved mouse back skin 1 h before IMQ treatment once a day for 4 days. Vehicle without DOB was applied to control mice.

*Intraperitoneal Injection of ADRB Antagonists*: For inhibiting ARβ1, ARβ1 antagonist ACE (0.2 mg, 10 mg kg^−1^ body weight) in 1 ml PBS or 1 ml PBS was intraperitoneally injected into each mouse 1 h before IMQ treatment once a day for 4 days. For inhibiting ARβ2, ARβ2 antagonist zenidolol hydrochloride (ZEN, 0.1 mg, 5 mg kg^−1^ body weight, MedChemExpress #HY‐13951) in 1 ml PBS or 1 ml PBS was intraperitoneally injected into each mouse 1 h before IMQ treatment once a day for 4 days. On day 5, the mice were sacrificed. Blood and skin samples were harvested for analysis.

### Cell Lines

SH‐SY5Y were purchased from National Collection of Authenticated Cell Culture (Shanghai, China). The cell line had recently undergone STR authentication and been tested for mycoplasma contamination. SH‐SY5Y cells were cultured in Dulbecco's modified Eagle medium (DMEM, HyClone #SH30022.01) supplemented with 10% FBS and 1% penicillin‐streptomycin solution. For experiments, cells were cultured in medium containing 5% FBS, 1% penicillin‐streptomycin solution, and 1.6 nm phorbol myristate 13‐acetate (PMA, Sigma Aldrich #P8139) for 8 days. When the cell density reached 70–80%, according to the instructions provided with the Lipofectamine 3000 kit (Thermo Fisher #L3000‐015), small interfering (si)CAMK2G (5′‐GGCUCAAUGUCCACUAUCATT‐3′) was transfected for *CAMK2G* knockdown. The control group was transfected with scrambled control. After 48 h, 100 µm 6‐OHDA (diluted with 0.2% ascorbic acid solution) was added to fresh complete medium; for the control group, only 0.2% ascorbic acid solution was added. The samples were analyzed after 12 and 24 h culture. All siRNAs were designed by and purchased from Qingke Biotech Corporation (Beijing, China).

### MACS Sorting and Cell Treatment

Three to five mouse spleen tissue samples were pooled to create samples subjected to magnet‐activated cell sorting. After surface staining with biotinylated antibodies against mouse T‐cell receptor (TCR) γ/δ (Biolegend #118 103), the cells were incubated with streptavidin microbeads (Biolegend #480 015). Primary γδT cells were then isolated magnetically using column‐free immunomagnetic separation. The cell population was sorted to >90% purity. The sorted **γ**δT cells were stimulated with 5 µm DOB or ACE for 1 h and then cultured in the same medium with interleukin (IL)−23 (25 ng ml^−1^, Novoprotein #CI18) for 4 h. The cells were then harvested for protein and RNA extraction. The MACS‐sorted γδT cells were stimulated or not with NE (1 µm), SB202190 (10 µm, MedChemExpress #HY‐10295), or SC75741 (10 µm, MedChemExpress # HY‐10496) for 1 h and activated with IL‐23 (25 ng ml^−1^) for 24 h. The cells were the harvested for intracellular staining. CD4^+^ T cells and CD8^+^ T cells were sorted using MojoSort Mouse CD4 Nanobeads (Biolegend, #480 070) and MojoSort Mouse CD8a Selection Kit (Biolegend, # 480 136). The cell population was sorted to >90% purity. Sorted mouse cells were used to extract RNA.

### Isolation of Mouse Skin Immune Cells

The isolation method of Yang et al.,^[^ ^55^ ^]^ was adopted with modification. The skin was minced fully and digested in DMEM containing bovine serum albumin (40 mg ml^−1^; Biofroxx #4240GR100), type 1 collagenase (4 mg ml^−1^, #C0130; Sigma), type 4 collagenase (2 mg ml^−1^, Worthington #LS004189), and hyaluronidase (2 mg ml^−1^, Sigma #H3506) at 37 °C and 225 rpm for 1.5 h. Then, DNase I (5 µg ml^−1^, Yuanye #S10073) was added to continue digestion for 30 min. The cell suspension was filtered through a 70‐µm cell strainer (Corning #431 751) and the skin immune cells were isolated with 40% and 80% lymphocyte separation solutions at 2000 rpm for 20 min. The cells were washed and resuspended in PBS for counting.

### Flow Cytometry

Single‐cell suspensions in PBS were pre‐incubated with Fc Block (anti‐mouse CD16/32, Biolegend #156 603) and then stained with DAPI solution (BD Pharmingen #564 907) and surface antigens. The fluorochrome‐conjugated surface antigens used for mouse skin immune cells were fluorescein isothiocyanate (FITC)‐CD45 (1/100, eBioscience #11‐0451‐82), R‐phycoerythrin (PE)–cyanine 7‐CD11b (1/100, BD Pharmingen #552 850), brilliant violet (BV) 605–lymphocyte antigen 6 complex, locus G (Ly‐6G; 1/100, BD Pharmingen #563 005), PE–Ly‐6C (1/100, BD Pharmingen #560 592), PE‐CF594‐CD11C (1/100, BD Pharmingen #562 454), BV510‐TCRγ/δ (1/100, Biolegend #118 131), peridinin chlorophyll protein complex (PerCP)/cyanine 5.5–TCRβ (1/100, Biolegend #109 228), and antigen‐presenting cell–CD3 (1/100, BD Pharmingen #553 066). The surface antigens used for human skin immune cells were PE‐TCRγ/δ (1/100, BD Pharmingen #555 717), PerCP/cyanine 5.5–TCRβ (1/100, BD Pharmingen #555 717), and BV510‐CD3 (1/100, BD Horizon #564 713). Then cells were fixed and permeabilized using fixation/permeabilization concentrate and diluent solutions (Invitrogen #00‐5523‐00) and then incubated with intracellular antibodies: PE–IL‐17A (1/100, BD Pharmingen #559 502) for mouse samples and anti–Adrenergic Receptor β1 (1/200, Invitrogen #PA1‐049) and FITC‐conjugated goat anti‐rabbit immunoglobulin (Ig)G H&L (1/100, ZSGB‐Bio #ZF‐0312) for human samples. The samples were analyzed using a CytoFLEX flow cytometer (Beckman Coulter, USA) and the CytExpert software (version 2.4).

### RNA Isolation and Quantitative PCR

Whole skin tissue samples were homogenized in TRIzol reagent (Ambion Life #15 596 018). Total RNA was isolated with TRIzol and reverse transcribed to cDNA using the Evo M‐MLV RT kit with gDNA Clean for qPCR (Accurate Biology #AG11728) according to the manufacturer's instructions. Real‐time quantitative PCR was performed using the SYBR Green Premix Pro Taq HS qPCR kit (Accurate Biology #AG11701) and a 7900HT fast real‐time PCR system (Thermo Fisher). Gene expression was quantified using the 2^−∆∆CT^ method and normalized to the housekeeping gene GAPDH. All primers, listed in Table S5 (Supporting Information), were acquired from Qingke Biotech Corporation (Beijing, China).

### Western Blotting

Skin‐tissue protein extracts were made by homogenizing samples, and the cells were lysed in RIPA lysis buffer (Beyotime #P0013B) supplemented with a protease and phosphatase inhibitor cocktail (Beyotime #P1048). Protein contents were quantified with a bicinchoninic acid protein assay kit (Thermo Scientific #A53225). Equal amounts (20 µg protein per well) of cell or tissue samples were separated on sodium dodecyl‐sulfate polyacrylamide gel electrophoresis gels (Beyotime, Shanghai, China), transferred to nitrocellulose membranes (GE #10 600 002), blocked in blocking buffer (Beyotime #P0252), probed with primary antibodies, and then incubated with horseradish peroxidase–conjugated secondary antibodies and detected with an electrochemiluminescence system (Thermo Fisher #34 095). The primary antibodies used were anti‐CaMKIIγ (1/500, Santa Cruz #sc‐517278), anti‐pCaMKII (1/2000, CST #12716T), anti‐ARRB1 (1/2000, Invitrogen #PA1‐049), anti‐tyrosine hydroxylase (TH, 1/200, CST #13106S), anti‐pTH (1/2000, Thermo Fisher #36‐8600), and anti–β‐actin (1/2000, CST #4970S). Anti‐pCaMKII was used due to the lack of specific phosphorylated CaMK2γ antibody on the market. The secondary antibodies used were goat anti‐rabbit IgG H&L (1/10000, Beyotime #A0208) and goat anti‐mouse IgG H&L (1/10000, Beyotime #A0216).

### Histological and Immunohistochemical Assays

Skin tissue samples were first processed to obtain several 4 × 4‐mm sections. Portions were fixed successively in 4% paraformaldehyde, embedded in paraffin and sectioned for hematoxylin and eosin staining and immunohistochemical (IHC) analysis. Other portions were fixed in 30% sucrose for frozen section staining and immunofluorescence. For the IHC analysis, paraffin‐embedded tissue sections were deparaffinized, heat retrieved at 100 °C for 15 min in 0.01 m sodium citrate buffer (pH 6.0), placed in 3% H_2_O_2_ for endogenous peroxidase blocking, blocked in blocking solution and then incubated with primary antibodies. Staining was performed using a 3,3′‐diaminobenzidine substrate kit (ZSGB‐Bio #ZLI‐9017) followed by hematoxylin counterstaining. Images were acquired under an Olympus BX53 upright microscope and analyzed using the CellSens software (version 1.5).

### Whole‐Mount Immunofluorescence

Skin tissue samples were first processed to obtain several 4 × 4‐mm sections. Portions were successively fixed in 4% paraformaldehyde, washed with PBS, immersed in 30% sucrose overnight at 4 °C, embedded in OCT, and made into 50‐µm thick sections. Sections were washed in PBS with 0.1% Triton and 0.2% BSA and incubated with 0.3 m glycine for 15 min. Sections were then washed, blocked (5% Goat serum; 0.5% BSA, 0.3% Triton, and 1% Fc block in PBS) overnight at 4 °C, incubated with primary antibodies overnight at 4 °C, and then incubated with secondary antibodies and fluorescence‐labelled second antibodies overnight at 4 °C, followed by incubation with DAPI (Beyotime #C1005) to stain the nuclei. Primary antibodies used include anti‐Tyrosine Hydroxylase (1/200, CST #13106S) and anti‐β3‐Tubulin (1/200, CST #5568). Secondary antibodies used include AlexaFluor 488‐conjugated goat anti‐rabbit IgG H&L (1/1000, Abcam #ab150077) and AlexaFluor647 ‐conjugated goat anti‐rabbit IgG H&L (1/1000, Abcam # ab150079). Fluorescence‐labelled second antibodies used include FITC anti‐CaMKIIγ (1/200, FabGennix #CaMK32‐FITC), FITC anti‐Mouse TCRγ/δ (1/100, BD Pharmingen #553 177), and PerCP/Cyanine5.5 anti‐Mouse TCRβ (1/100, Biolegend #109 228). Images were obtained under a confocal scanning microscope (TCS SP8; Leica) and analyzed using the LAS X software (version 3.5.1.18803).

### Enzyme‐Linked Immunosorbent Assay

For blood neurotransmitter measurement, venous blood was collected from all subjects and placed in a refrigerator at 4 °C for 30–60 min. After coagulation, the blood was centrifuged for 15 min at 3000 rpm. The supernatant was collected. For skin neurotransmitter measurement, full‐thickness skin samples (25 mg) were collected and homogenized in 250 µl 1 × PBS. The homogenized mixture was centrifuged for 15 min at 12 000 rpm and the supernatant was collected. Neurotransmitter concentrations were determined using enzyme‐linked immunosorbent assay kits for mouse NE (Meimian #MM‐0876M1) and human NE (Abnova #KA1891) according to the manufacturers’ protocols.

### RNA Sequencing

Total RNA was extracted from whole mouse skin. A cDNA library (300 ± 50 bp) was obtained from ≈1 µg of total RNA after quality control. Then, a sequencing library was assembled using an Illumina Novaseq 6000 device and 2 × 150‐bp paired‐end sequencing chemistry. The statistical significance of genes was defined by >2‐fold changes or <0.5‐fold change and *p *< 0.05. Gene ontology and Kyoto Encyclopedia of Genes and Genomes pathway enrichment analyses of differentially expressed genes were performed. Transcript per million values for all sequenced genes were used to generate heat maps. RNA sequencing was conducted by the staff at LC Biotech Corporation (Hangzhou, China).

### 10x Genomics Single‐Cell RNA Sequencing

Skin tissues from VAS‐ and IMQ‐treated mice were dissociated and single‐cell suspensions were prepared for the construction of a 10x chromium genomics library and sequencing. 10x genomics single‐cell RNA sequencing was conducted by the staff at LC Biotech Corporation.

### 4D Label‐Free Quantitative Proteomics

To detect differential protein expression induced by DOB stimulation, DOB‐ and vehicle‐treated **γ**δT cells were collected for 4D label‐free quantitative proteomics analysis. The analysis was conducted by the staff at LC Biotech Corporation.

### Screening of Small Molecule Inhibitors

Compounds from a natural product library and a drug library with affinity less than −11.5 kcal mol^−1^ and from the ChemDiv with affinity less than ‐14 kcal mol^−1^ were selected and had a Lipinski's rule of five score greater than 0.5, a central nervous system drugs (CNS) score greater than 0.1, and a blood‐brain barrier (BBB) score greater than −1, finally 678 compounds were obtained. Based on the 678 compounds, compounds with poor druggability were further screened, such as compounds with topological polar surface area (TPSA)>70, compounds with poor intestinal absorption (Health Impact Assessment Category ‐), compounds easily hydrolyzed by metabolic enzymes (2D6 value = very high), and compounds that might had cardiotoxicity (hERG>7), and compounds with the molecular weight greater than 500. Finally, 570 compounds were retained. Based on the structural similarity (threshold = 0.7), the 570 compounds were clustered into 70 groups. In each group, compounds with high affinity were selected to form compounds with high affinity and diversity, totaling 293 compounds. The above 293 compounds were the final compounds screened in this project, and their indexes had met the requirements of druggability. To further identify possible active compounds, subsequent activity tests were conducted on the compounds with scoring functions top100 (ChemDiv database) and top30 (TargetMol® database). 130 (TOP30, TOP100) small molecule inhibitors of CaMK2γ were screened according to the order of affinity using Biacore surface plasmon resonance. The compounds with inhibitory effect and higher safety were screened out using CaMK2γ Kinase Assay (Promega #V9201).

### Statistical Analyses

The GraphPad Prism 8 software was used for all statistical analyses. Data were presented as means ± standard deviations. Means were compared between groups using the two‐tailed *t* test and among groups using one‐and two‐way analyses of variance. The significance level was set to *p < s0.05, **p < 0.01, ***p < 0.001; ns, not significant.

### Study Approval

All animal experiments were approved by the Laboratory animal ethics committee of Anhui Medical University (Approval number: LLSC20190208) and conformed to the guidelines outlined in the Guide for the Care and Use of Laboratory Animals. All efforts were made to minimize suffering. Human subjects’ study was approved by the Biomedical ethics committee of Anhui Medical University (Approval number: Quick‐PJ 2022‐11‐42) and were performed in accordance with the principles of the Declaration of Helsinki, Ethics review on biomedical research involving human subjects and International ethical guidelines for biomedical research involving.

## Conflict of Interest

The authors declare no conflict of interest.

## Author Contributions

Y.Y., W.C., and B.L. contributed equally to this work. L.D.S. conceived the studies and supervised the research. L.D.S., Y.F.Y., Q.Z., and B.L. designed the studies. Y.F.Y. and Q.Z. conducted experiments and analyzed data. Z.L., Y.R.W., W.C.F., and Y.M.B. fed and identified mice. W.W.C. and Y.W.M. coordinated clinical investigation and collected clinical samples. H.H.B. provided experimental mice and revised the manuscript. Y.F.Y., B.L. and L.D.S. wrote the manuscript. All authors reviewed the manuscript.

## Supporting information

Supporting Information

## Data Availability

The data that support the findings of this study are available from the corresponding author upon reasonable request.
