# Supplementary material for: Cutaneous Calcium/Calmodulin‐Dependent Protein Kinase II‐γ–Positive Sympathetic Nerves Secreting Norepinephrine Dictate Psoriasis
Source: Adv Sci (Weinh). 2024 Mar 28;11(23):2306772. doi: 10.1002/advs.202306772 (PMC11187923; doi:10.1002/advs.202306772)
Supplement: Supplementary file 1 — Supporting Information [file ADVS-11-2306772-s001.pdf]

ADVANCED SCIENCE
Open Access

Supporting Information

for *Adv. Sci.*, DOI 10.1002/advs.202306772

Cutaneous Calcium/Calmodulin-Dependent Protein Kinase II-γ–Positive Sympathetic Nerves Secreting Norepinephrine Dictate Psoriasis

Yafen Yu, Weiwei Chen, Bao Li, Zhuo Li, Yirui Wang, Yiwen Mao, Wencheng Fan, Yuanming Bai, Hongbo Hu, Qi Zhen and Liangdan Sun**

Supporting information

Cutaneous CAMK2γ$^+$ sympathetic nerves secreting norepinephrine dictates psoriasis

Yafen Yu[#], Weiwei Chen[#], Bao Li[#], Zhuo Li, Yirui Wang, Yiwen Mao, Wencheng Fan, Yuanming Bai, Hongbo Hu, Qi Zhen [*] and Liangdan Sun[*]

Supplementary Figures

Figure. S1

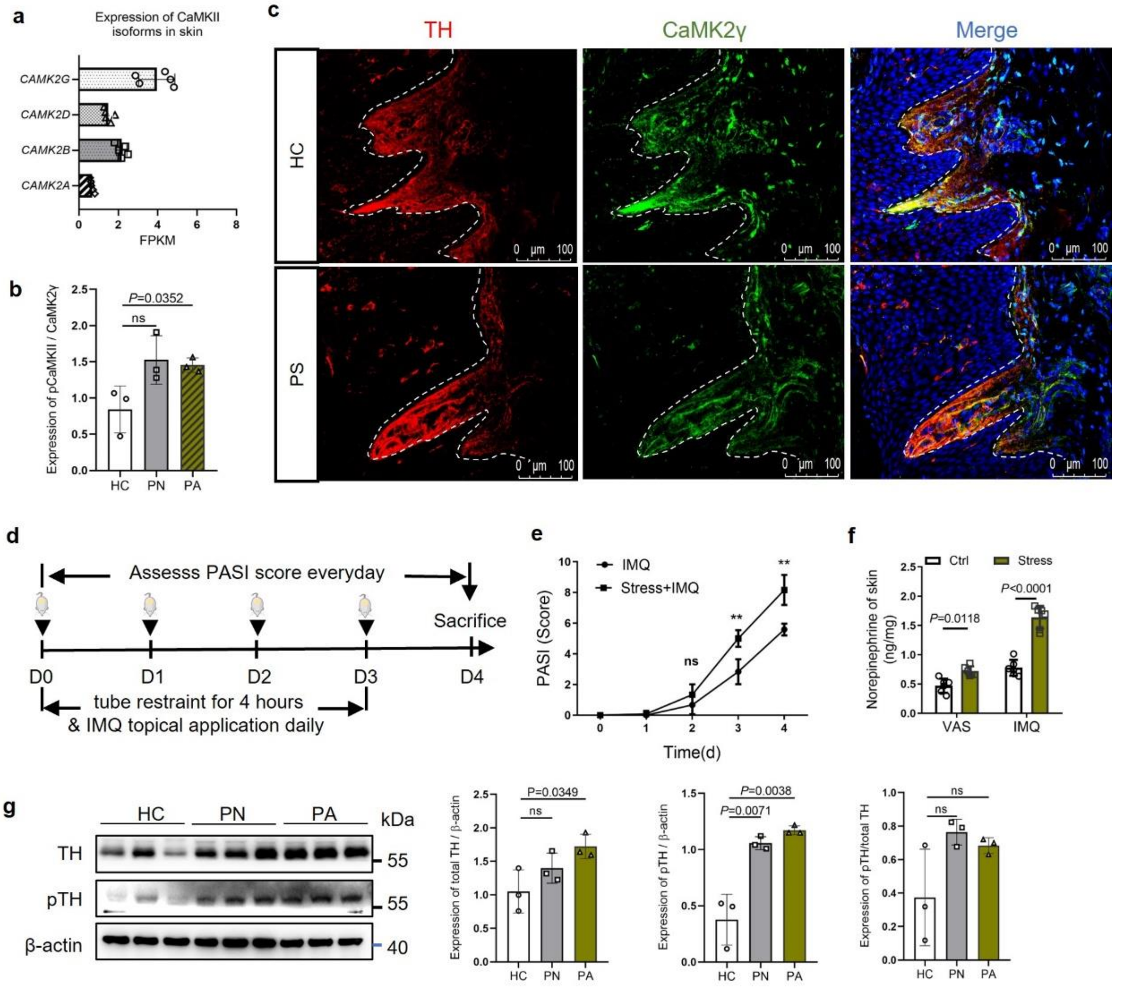

Figure S1. Cutaneous CaMK2γ expression and stress mouse model construction. **(a)** Expression of CAMKII family genes in WT mouse skin, determined by RNA-seq (n = 5). **(b)** Quantification of CaMK2γ and pCAMKII in skin from healthy control, non-lesional and psoriatic lesional skin. **(c)** Representative immunofluorescent images of skin sections from healthy controls and psoriatic lesions (n = 3) stained for TH (red), CaMK2γ (green) and DAPI (blue). Scale bars = 100 μm. **(d)** Flow chart of acute stress induction in the mouse model. **(e)** PASI scores (n = 5). **(f)** NE concentrations in mouse skin, determined by enzyme-linked immunosorbent assay. **(g)** Western blot and

quantification of TH and pTH in skin from healthy control, non-lesional and psoriatic lesional skin. Data are representative of three independent experiments and shown as mean ± SD. (b, e, g) two-tailed unpaired Student's t test. (f) Two-way analysis of variance. *$p < 0.05$, **$p < 0.01$, ***$p < 0.001$; ns, not significant.

Figure. S2

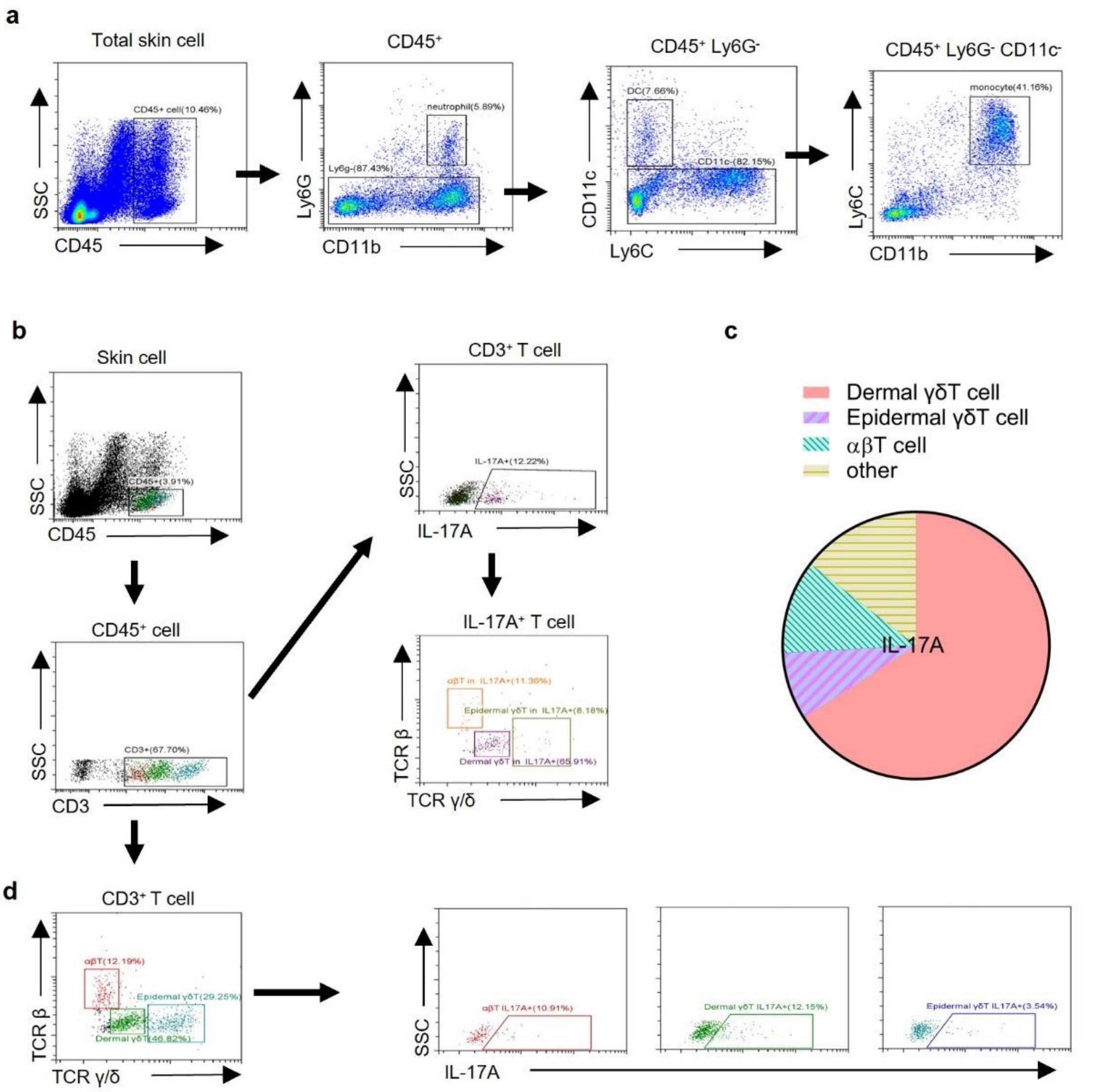

Figure S2. Flow cytometric analysis of inflammatory infiltrating cells in digested murine skin. **(a)** Representative flow cytometric plots of the gating strategies for monocytes, neutrophils, and dendritic cells in digested murine skin. **(b)** IL-17A$^+$ T cells were gated into dermal γδT17 cells, epidermal γδT17 cells and αβT17 cells. **(c)** Relative frequencies among total IL-17A$^+$ cells. **(d)** Gating strategy to identify IL-17A$^+$ cells among dermal γδT cells, epidermal γδT cells and αβT cells.

Figure. S3

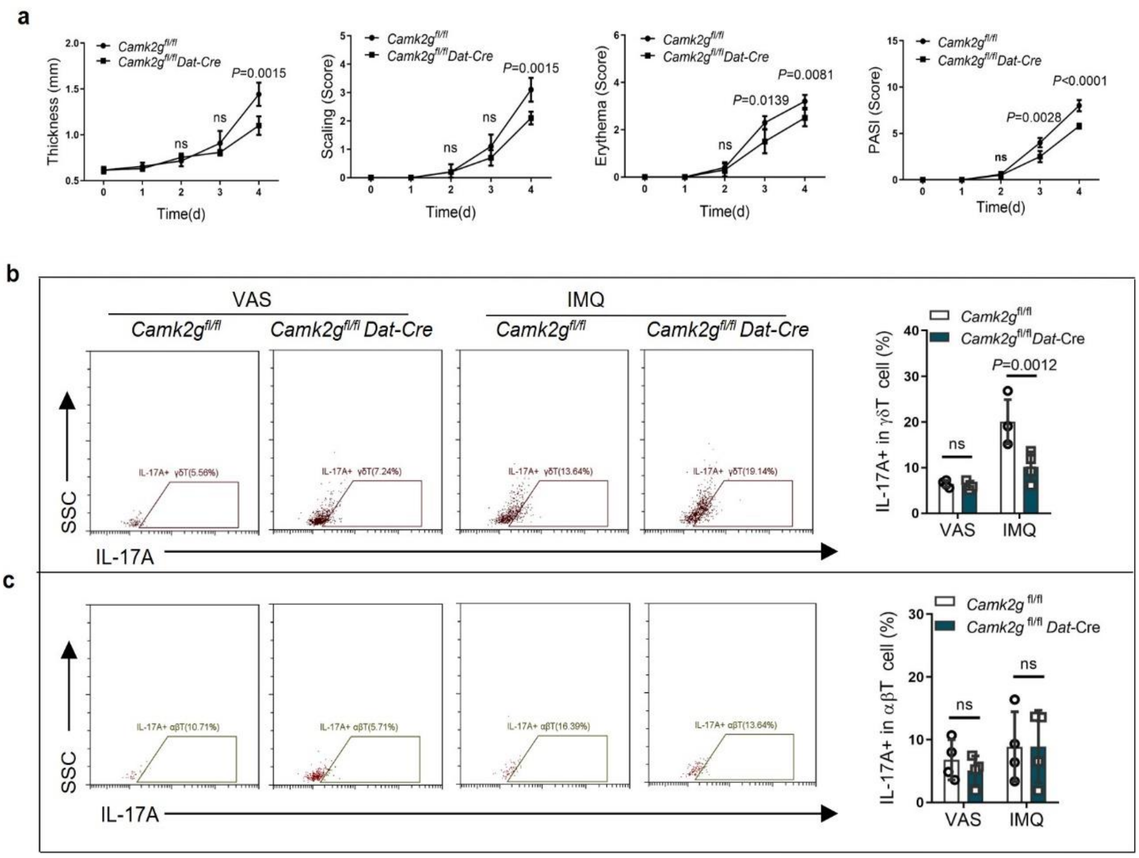

Figure S3. Data from IMQ-treated *Camk2g*$^{fl/fl}$ and *Camk2g*$^{fl/fl}$ *Dat-Cre* mice. (a) Scoring curves of back skin thickness, scaling, and erythema (n = 5). **(b, c)** Representative flow cytometric images and statistics for IL-17A^{+} γδT cells (b) and αβT cells (c) from digested back skin. Data are representative of three independent experiments and shown as mean ± SD. (a) two-tailed unpaired Student's t test. (b, c) Two-way analysis of variance. *$p < 0.05$, **$p < 0.01$, ***$p < 0.001$; ns, not significant.

Figure. S4

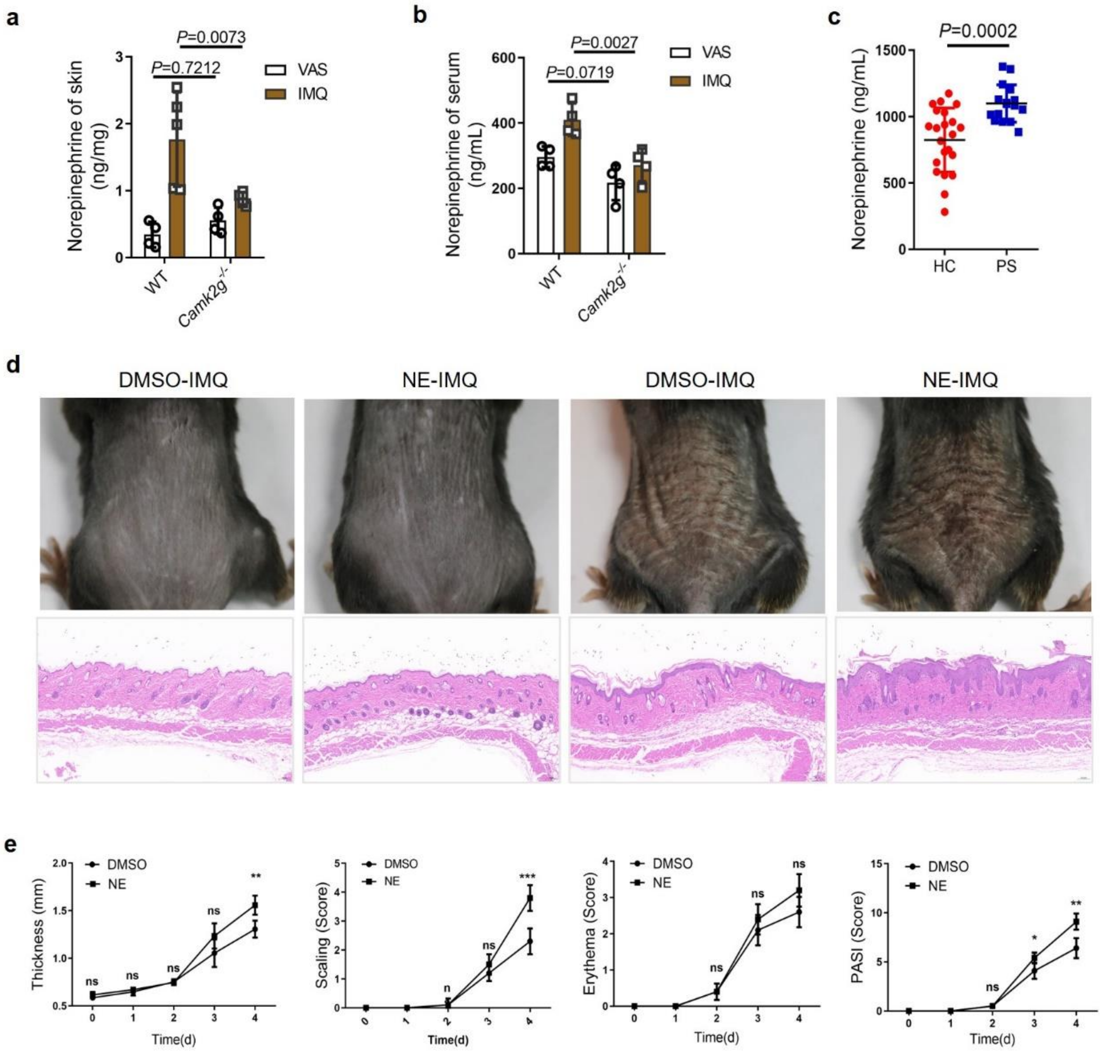

Figure S4. NE concentrations and mouse model of IMQ-induced psoriasis with NE injection. (a) Skin NE concentrations in mice on day 4 of IMQ treatment (n = 4). **(b)** Serum NE concentrations in mice on day 4 of IMQ treatment (n = 4). **(c)** Serum NE concentrations in healthy controls (*n* = 22) and patients with psoriasis (*n* = 18). **(d)** Representative photographs and HE–stained sections of back skin on day 4 of IMQ treatment (n = 5). Scale bars = 50 μm. **(e)** Scoring curves of back skin thickness, scaling, and erythema (n = 5). Data are representative of three independent experiments and shown as mean ± SD. (a, b) Two-way analysis of variance. (c, e) Two-tailed unpaired Student's t test. *$p < 0.05$, **$p < 0.01$, ***$p < 0.001$; ns, not significant.

Figure. S5

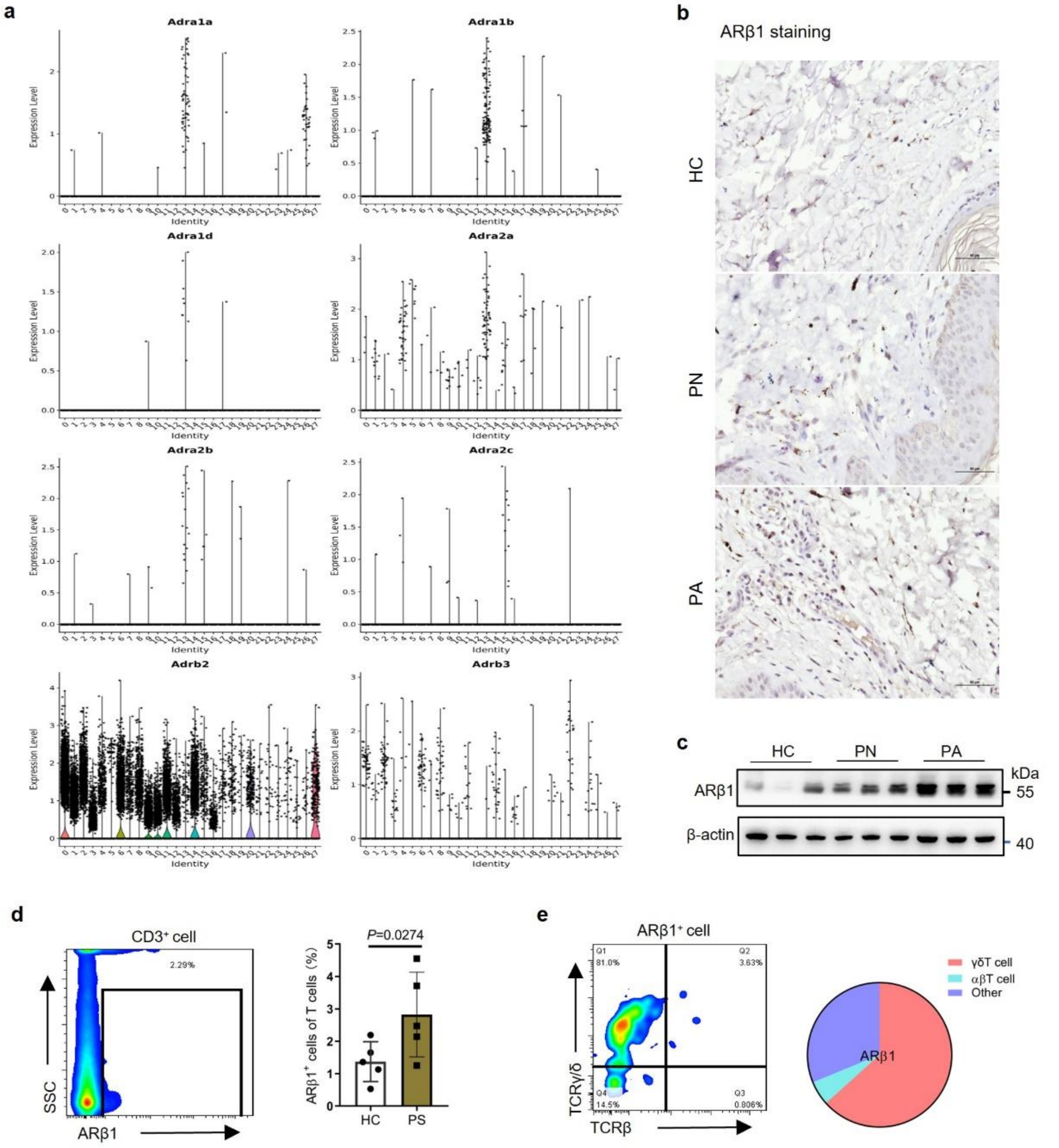

Figure S5. ARβ1 expression patterns in the mouse model and patients. (a) Expression of adrenergic receptors in VAS-treated control and IMQ-treated psoriatic murine skin (10X single cell sequencing). **(b)** Representative immunohistochemical images of human psoriatic lesional skin and healthy skin stained for ARβ1 (n = 3). Scale bars = 20 μm. **(c)** Western blots of ARβ1 in healthy controls, psoriatic non-lesional and lesional skin (n = 3). **(d)** Frequencies of ARβ1$^+$ T cells in human psoriatic lesional skin

and healthy skin, determined by flow cytometry (*n*=*5*). **(e)** Frequencies of ARβ1$^+$ T-cell subsets from digested human skin, determined by flow cytometry. Data are representative of three independent experiments and shown as mean ± SD. (d) Two-tailed unpaired Student's t test.

Figure. S6

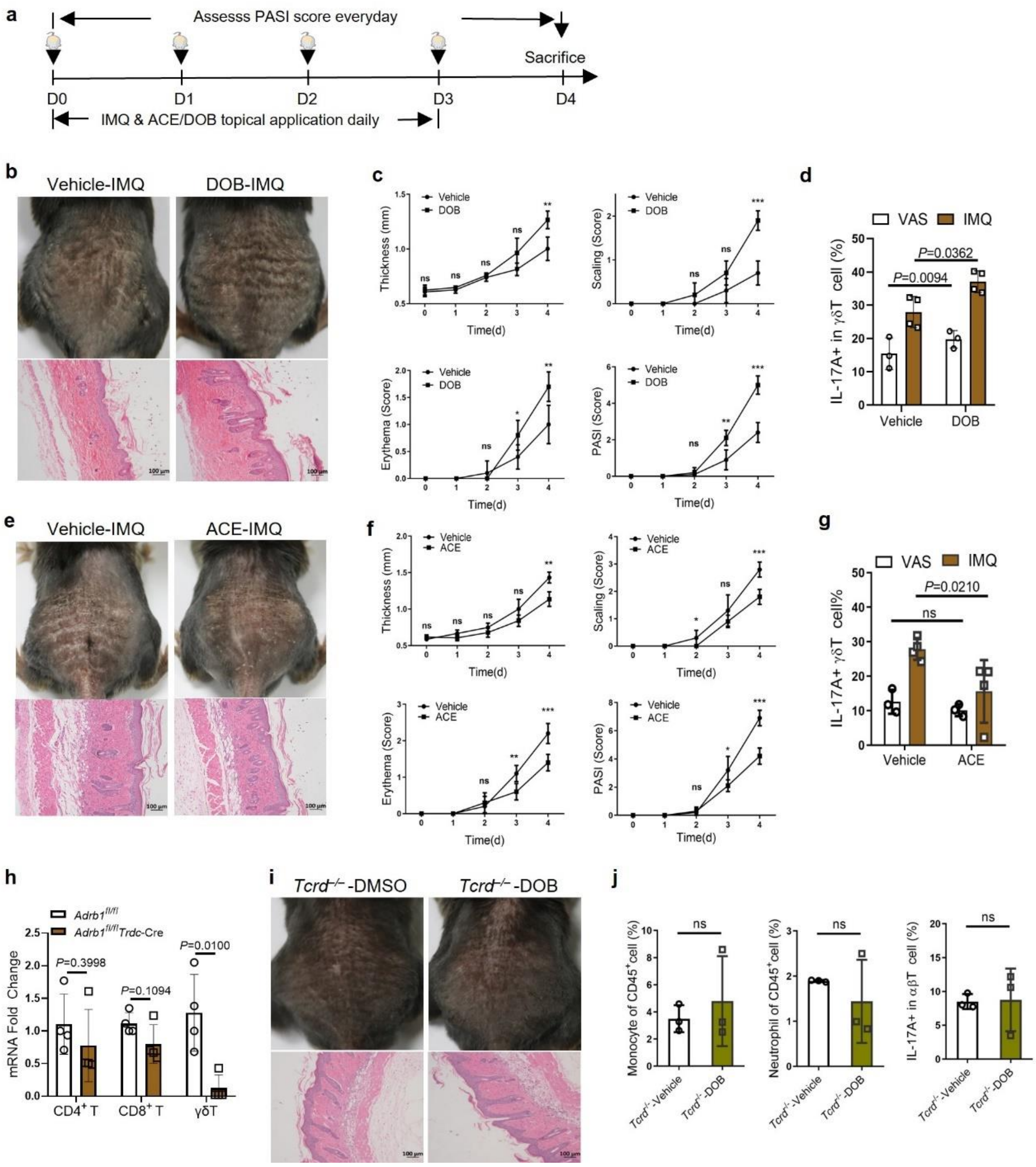

Figure S6. Mouse model of IMQ-induced psoriasis with ARβ1 agonist and antagonist application. **(a)** Flow chart of topical ACE and DOB application in IMQ-induced mouse model. **(b)** Representative photographs and HE–stained sections of back skin from vehicle- and DOB-treated mice on day 4 of IMQ treatment. Scale bars = 100 µm. **(c)** Scoring curves of back skin thickness, scaling, and erythema (n = 5) (n=5). **(d)** Frequencies of IL-17A^{+} γδT cells from digested back skin, determined by flow

cytometry (n = 4). **(e)** Representative photographs and HE–stained sections of back skin from vehicle- and ACE-treated mice on day 4 of IMQ treatment (*n* = 5). Scale bars=100 μm. **(f)** Scoring curves of back skin thickness, scaling, and erythema (n = 5) (n=5). **(g)** Frequencies of IL-17A$^+$ γδT cells from digested back skin, determined by flow cytometry (n = 4). **(h)** Quantitative PCR analysis of *Adrb1* expression in MACS-sorted CD4$^+$ T cells, CD8$^+$ T cells and γδT cells from mouse spleen. **(i, j)** *Tcrd*$^{-/-}$ mouse model of IMQ-induced psoriasis with ARβ1 agonist application (*n* = 5). **(i)** Representative photographs and HE–stained sections of back skin vehicle- and DOB-treated mice on day 4 of IMQ treatment. Scale bars = 100 μm. **(j)** Total inflammatory neutrophils and monocytes, and frequency of IL-17A$^+$ T cells from digested back skin on day 4 of IMQ treatment, determined by flow cytometry. Data are representative of three independent experiments and shown as mean ± SD. (c, f, h, j) Two-way analysis of variance. (d, g) Two-tailed unpaired Student's t test. *$p < 0.05$, **$p < 0.01$, ***$p < 0.001$; ns, not significant.

Figure. S7

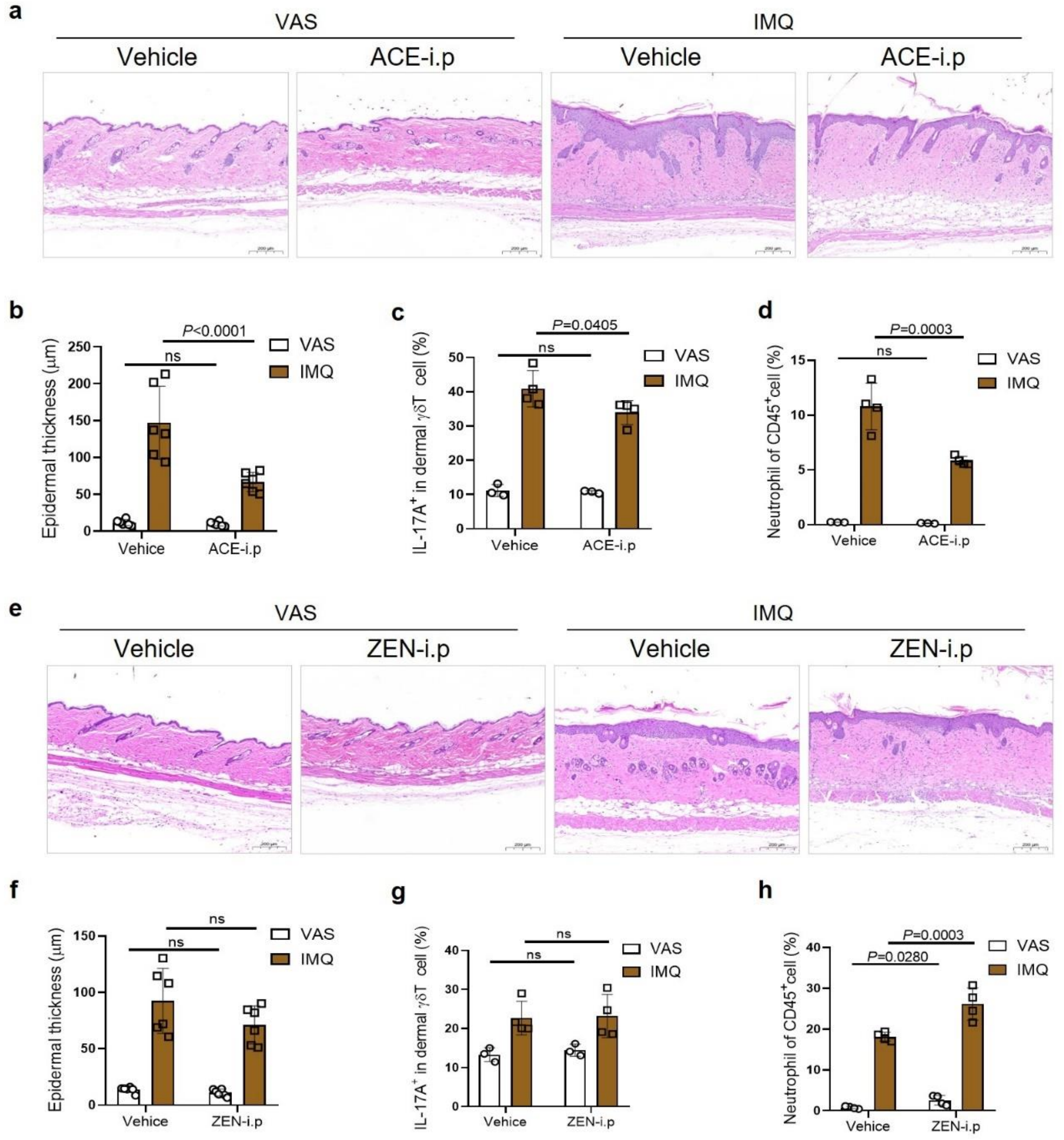

Figure S7. Mouse model of IMQ-induced psoriasis with intraperitoneal injection of ARβ1 and ARβ2 antagonist. (a) Representative HE–stained sections of back skin from vehicle- and ACE-treated mice on day 4 of IMQ treatment. Scale bars = 100 μm. **(b)** Statistical analysis of epidermal thickness. **(c)** Frequencies of IL-17A+ γδT cells from digested back skin, determined by flow cytometry (n = 4). **(d)** The percentage of total inflammatory neutrophils among $CD45^+$ cells from digested back skin (n = 4) on day 4. **(e)** Representative HE–stained sections of back skin from vehicle- and ZEN-

treated mice on day 4 of IMQ treatment. Scale bars = 100 μm. **(f)** Statistical analysis of epidermal thickness. **(g)** Frequencies of IL-17A+ γδT cells from digested back skin, determined by flow cytometry (n = 4). **(h)** The percentage of total inflammatory neutrophils among CD45+ cells from digested back skin (n = 4) on day 4. Data are representative of three independent experiments and shown as mean ± SD. (b-d, f-h) Two-way analysis of variance. ns, not significant.

Figure. S8

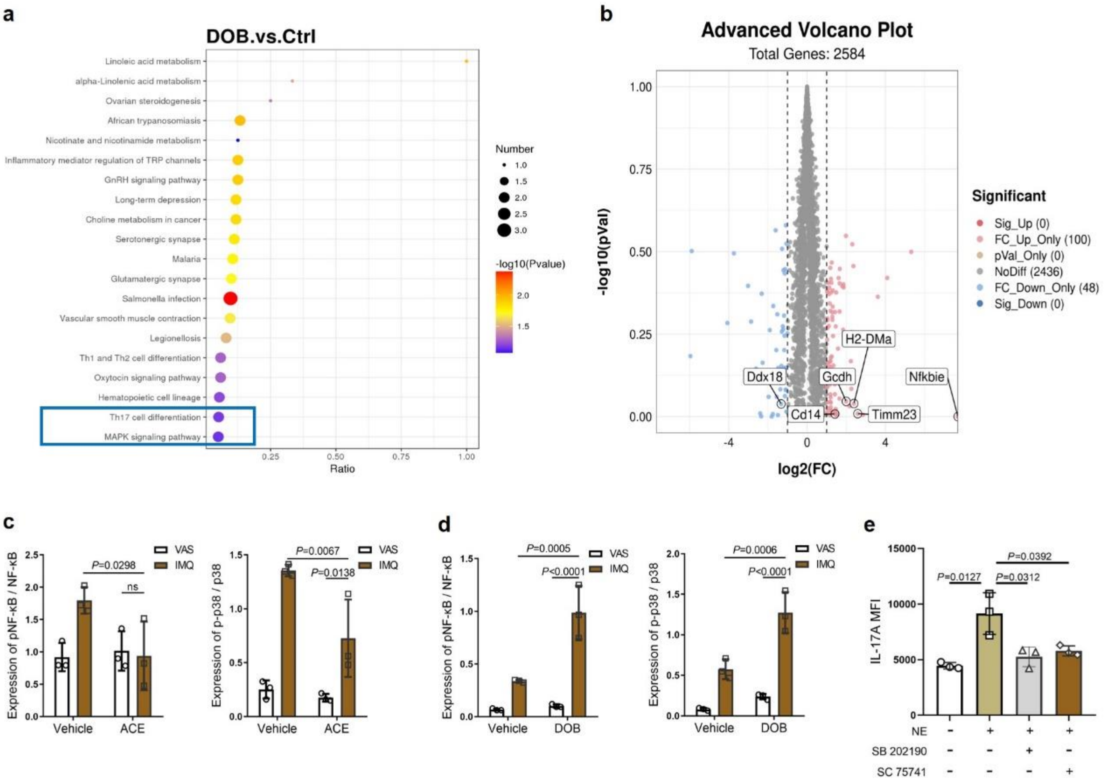

Figure S8. MACS-sorted γδT cells and mouse model of IMQ-induced psoriasis with ARβ1 agonist and antagonist application. (a, b) 4D label-free quantitative proteomics of DOB- and vehicle-treated γδT cells. Kyoto Encyclopedia of Genes and Genomes pathway enrichment (a) and volcano plot (b) of differentially expressed proteins. **(c, d)** Quantification of pNF-κB / NF-κB and p-p38 / p38 in back skin from ACE- (c) and DOB-treated (d) mice on day 4 of IMQ treatment. **(e)** Statistical analysis of the MFI of IL-17A in IL-23-activated γδT cells. Data are representative of three independent experiments and shown as mean ± SD. (c, d) Two-way analysis of variance. (e) Two-tailed unpaired Student's t test. ns, not significant.

Figure. S9

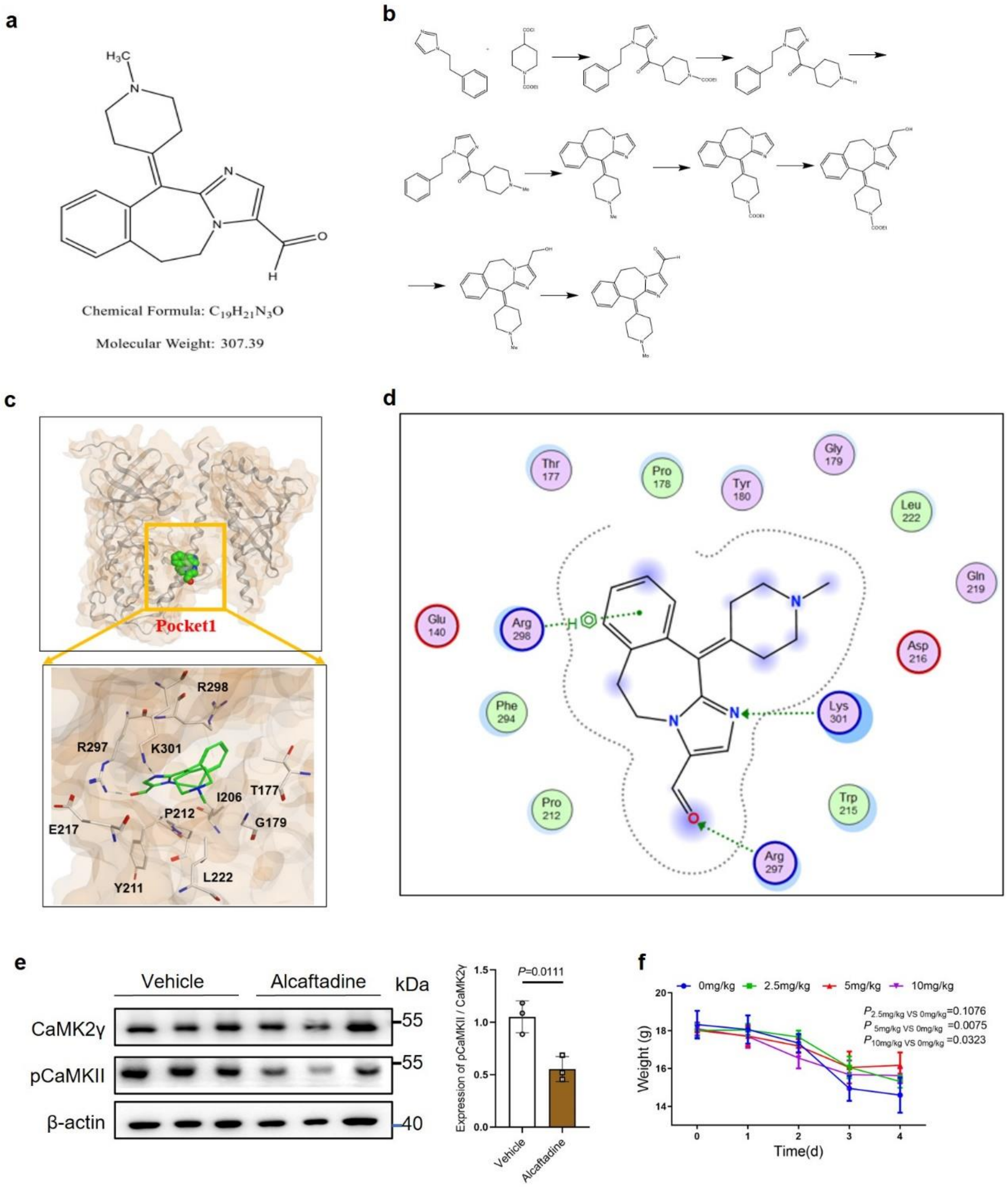

Figure S9. Data of alcaftadine. (a) Chemical structure. **(b)** Synthetic routes. **(c, d)** Diagrams of interaction between alcaftadine and CaMK2γ. **(c)** Structure of the complex formed by human CaMK2γ (orange) and alcaftadine (green). The lower portion shows interaction of compounds in the pocket 1 region of CaMK2γ, where R297, R298 and K301 form hydrogen-bond interactions with alcaftadine. **(d)** 2D interaction pattern of

alcaftadine and interacting amino acids at the target site, where R297, R298 and K301 form hydrogen bonds with alcaftadine. **(e)** Western blot and quantification of pCaMKII and total CaMK2γ in back skin from vehicle- and alcaftadine -treated mice on day 4 of IMQ treatment. **(f)** Statistical analysis of mouse body weight. Data are representative of three independent experiments and shown as mean ± SD. (e, f) Two-tailed unpaired Student's t test. ns, not significant.

Supplementary Table

Table S1. Exclusion criteria for patient with psoriasis and healthy control.

patient with psoriasis	healthy control
1）Patients with severe medical conditions leading to poor physical health, such as lack of autonomy, coagulopathy, severe anemia, and tumors.<br>2）Hemolyzed blood before the experiment.<br>3）Patients who have been enrolled more than once.<br>4）Systematic use of drugs (immune-modulators and immunosuppressants) within one month, and external use of drugs in rash sites within half a month;<br>5）Refused to sign the informed consent for the study.	1）Patients with severe medical conditions leading to poor physical health, such as lack of autonomy, coagulopathy, severe anemia, and tumors.<br>2）Persons diagnosed with autoimmune disease, systemic disease and any reported family history of psoriasis (including first -, second -, and third-degree relatives).<br>3）Hemolyzed blood before the experiment.<br>4）Refused to sign the informed consent for the study.

Table S2. Sample information of serum for ELISA.

Sample No.	Diagnose	Gender	Age (years)	Stage of disease	Sample type
20180625HFxUC140	Health control	Male	28	No	Peripheral blood
20180625HFxUC148	Healthy control	Male	26	No	Peripheral blood
20180625HFxUC150	Healthy control	Male	22	No	Peripheral blood
20180625HFxUC158	Healthy control	Male	38	No	Peripheral blood
20180625HFxUC159	Healthy control	Male	26	No	Peripheral blood
20180625HFxUC3	Healthy control	Male	38	No	Peripheral blood
20180625HFxUC31	Healthy control	Male	30	No	Peripheral blood
20180625HFxUC35	Healthy control	Male	26	No	Peripheral blood
20180625HFxUC40	Healthy control	Male	46	No	Peripheral blood
20180625HFxUC50	Healthy control	Male	30	No	Peripheral blood
20180625HFxUC63	Healthy control	Male	31	No	Peripheral blood
20180625HFxUC64	Healthy control	Male	35	No	Peripheral blood
20180625HFxUC68	Healthy control	Male	25	No	Peripheral blood
20180625HFxUC74	Healthy control	Male	38	No	Peripheral blood
20180625HFxUC75	Healthy control	Male	36	No	Peripheral blood
20180625HFxUC77	Healthy control	Male	35	No	Peripheral blood
20180625HFxUC8	Healthy control	Male	22	No	Peripheral blood
20180625HFxUC80	Healthy control	Male	29	No	Peripheral blood
20180625HFxUC86	Healthy control	Male	27	No	Peripheral blood
20180625HFxUC91	Healthy control	Male	21	No	Peripheral blood
20180625HFxUC93	Healthy control	Male	31	No	Peripheral blood
20180625HFxUC97	Healthy control	Male	29	No	Peripheral blood
PAD0766	patient	Male	49	Stable	Peripheral blood
PAD0767	patient	Male	48	Active	Peripheral blood
PAD0780	patient	Male	29	Active	Peripheral blood

PAD0785	patient	Male	30	Stable	Peripheral blood
PAD0790	patient	Male	23	Active	Peripheral blood
PAD0794	patient	Male	45	Active	Peripheral blood
PAD0805	patient	Male	32	Active	Peripheral blood
PAD0812	patient	Male	19	Active	Peripheral blood
PAD0833	patient	Male	32	Stable	Peripheral blood
PAD0834	patient	Male	37	Active	Peripheral blood
PAD0837	patient	Male	24	Active	Peripheral blood
PAD0838	patient	Male	42	Active	Peripheral blood
PAD0839	patient	Male	18	Active	Peripheral blood
PAD0843	patient	Male	30	Active	Peripheral blood
PAD0856	patient	Male	18	Active	Peripheral blood
PAD0869	patient	Male	23	Active	Peripheral blood
PAD0875	patient	Male	28	Active	Peripheral blood
PAD0878	patient	Male	50	Active	Peripheral blood

Table S3. Sample information of skin section.

Sample No.	Diagnose	Gender	Age (years)	Sample type	Sampling location
HC2	Healthy control	Female	33	Skin	Back
HC3	Healthy control	Female	29	Skin	Back
HC11	Healthy control	Male	36	Skin	Back
HC12	Healthy control	Male	44	Skin	Back
HC13	Healthy control	Male	23	Skin	Back
PA1	patient	Female	40	Skin	Back
PA2	patient	Male	24	Skin	Back
PA3	patient	Male	32	Skin	Back
PA4	patient	Male	42	Skin	Back
PA5	patient	Female	26	Skin	Back

Table S4. Mouse skin thickness rating scale.

Thickness (μm)	0.60⩽ <0.80	0.80⩽ <1.00	1.00⩽ <1.20	1.20⩽ <1.40	1.40⩽ <1.60	1.60⩽ <1.80	1.80⩽ <2.00	2.00⩽ <2.20	⩾2.20
Score	0	0.5	1	1.5	2	2.5	3	3.5	4

Table S5. **Primer sequence of Quantitative PCR.**

Gene	Primers named	sequence (5’-3’)
GAPDH	hu-GAPDH-F	GTCTCCTCTGACTTCAACAGCG
	hu-GAPDH-R	ACCACCCTGTTGCTGTAGCCAA
CAMK2G	hu-*CAMK2G*-F	ACCCGTTTCACCGACGACTA
	hu-*CAMK2G*-R	CTCCTGCGTGGAGGTTTTCTT
NCF1	hu-NCF1-F	AATGGCAGGACCTGTCGGAGAA
	hu-NCF1-R	CCTGTTCTCTGGATTGATCGCC
NCF2	hu-NCF2-F	ACTACTGCCTGACTCTGTGGTG
	hu-NCF2-R	CCTCCACTTGGCTGCCTTTCTT
NOX2	hu-NOX2-F	CTCTGAACTTGGAGACAGGCAAA
	hu-NOX2-R	CACAGCGTGATGACAACTCCAG
TH	hu-Th-F	ACTGGTTCACGGTGGAGTTC
	hu-Th-R	AGCTCCTGAGCTTGTCCTTG
Gapdh	mo-Gapdh -F	GTGTTCCTACCCCCAATGTG
	mo-Gapdh-R	GGTCCTCAGTGTAGCCCAAG
Camk2g	mo-*CAMK2G*-F	GGACACAGTCACTCCTGAAGCT
	mo-*CAMK2G*-R	TCTACCGTCTCTTGGCGATGCA
Il17f	mo-Il17f-F	AACCAGGGCATTTCTGTCCCAC
	mo-Il17f-R	GGCATTGATGCAGCCTGAGTGT
Il22	mo-Il22-F	ATGAGTTTTTCCCTTATGGGGAC
	mo-Il22-R	GCTGGAAGTTGGACACCTCAA
Il1b	mo-Il1b-F	TGGACCTTCCAGGATGAGGACA
	mo-Il1b-R	GTTCATCTCGGAGCCTGTAGTG
Il6	mo-Il6-F	TACCACTTCACAAGTCGGAGGC
	mo-Il6-R	CTGCAAGTGCATCATCGTTGTTC
Tnfa	mo-Tnfa-F	ACTGGCAGAAGAGGCACTC
	mo-Tnfa-R	CTGGCACCACTAGTTGGTTG
Adrb1	mo-Adrb1-F	ACCACTGTGGACAGCGATTCG
	mo- Adrb1-R	TCTCTTCCTCTAGGGATGCAGAGC

hu: human qPCR primers.

mo: mouse qPCR primers
